# Frailty and outcomes in adults undergoing systemic anticancer treatment: a systematic review and meta-analysis

**DOI:** 10.1093/jnci/djaf017

**Published:** 2025-01-30

**Authors:** Jessica Pearce, Sally Martin, Sophie Heritage, Emma G Khoury, Joanna Kucharczak, Thitikorn Nuamek, David A Cairns, Galina Velikova, Suzanne H Richards, Andrew Clegg, Alexandra Gilbert

**Affiliations:** Leeds Institute of Medical Research at St James’s, University of Leeds, Leeds LS2 9JT, United Kingdom; Leeds Institute of Oncology, Leeds Teaching Hospitals National Health Service Trust, Leeds LS9 7TF, United Kingdom; Leeds Institute of Oncology, Leeds Teaching Hospitals National Health Service Trust, Leeds LS9 7TF, United Kingdom; Royal Papworth Hospital National Health Service Foundation Trust, Cambridge CB2 0AY, United Kingdom; Academic Cancer Sciences Unit, University of Southampton, Southampton SO16 6YD, United Kingdom; Department of Oncology, Cambridge University Hospitals, University of Cambridge, Cambridge CB2 0SP, United Kingdom; Clinical Oncology, The Christie National Health Service Foundation Trust, Manchester M20 4BX, United Kingdom; Leeds Cancer Research UK Clinical Trials Unit, University of Leeds, Leeds LS2 9JT, United Kingdom; Leeds Institute of Medical Research at St James’s, University of Leeds, Leeds LS2 9JT, United Kingdom; Leeds Institute of Oncology, Leeds Teaching Hospitals National Health Service Trust, Leeds LS9 7TF, United Kingdom; Leeds Institute of Health Sciences, University of Leeds, Leeds LS2 9NL, United Kingdom; Academic Unit for Ageing and Stroke Research, Bradford Institute for Health Research, Bradford BD9 6RJ, United Kingdom; Leeds Institute of Medical Research at St James’s, University of Leeds, Leeds LS2 9JT, United Kingdom; Leeds Institute of Oncology, Leeds Teaching Hospitals National Health Service Trust, Leeds LS9 7TF, United Kingdom

## Abstract

**Background:**

It is increasingly recognized that frailty should be assessed and considered in treatment decision making in patients with cancer. This review and meta-analysis synthesizes existing evidence evaluating the association between baseline frailty and systemic anticancer treatment outcomes in adults with cancer.

**Methods:**

Five databases were systematically searched from database inception to January 2023 to identify prognostic factor studies (cohort or case-control design) reporting the associations between validated frailty assessments (pretreatment) and follow-up outcomes in adults with solid-organ malignancy undergoing systemic anticancer treatment. Risk of bias was assessed via Quality of Prognosis Studies in Systematic Reviews tool. Where appropriate, associations between frailty and outcomes (survival, toxicity, treatment tolerance, functional decline/quality of life, and hospitalization) were synthesized in meta-analysis and presented as forest plots.

**Results:**

A total of 58 studies met inclusion criteria. They were undertaken in a range of tumor sites and mainly in older patients and advanced and/or palliative disease settings. Most had low or moderate risk of bias. Nine frailty assessment tools were evaluated. Four outcomes were synthesized in meta-analysis, which demonstrated the prognostic value of 2 tools: Geriatric-8 (survival, treatment tolerance, hospitalization) and Vulnerable Elders Survey-13 (survival, toxicity, treatment tolerance). Overall pooled estimates indicate that frailty conveys an increased risk of mortality (hazard ratio [HR] = 1.68, 95% confidence interval [CI] = 1.41 to 2.00), toxicity (odds ratio [OR] 1.83, 95% CI = 1.24 to 2.68), treatment intolerance (OR = 1.68, 95% CI = 1.32 to 2.12), and hospitalization (OR = 1.94, 95% CI = 1.32 to 2.83).

**Conclusion:**

Simple, brief frailty assessments including Geriatric-8 and Vulnerable Elders Survey-13 are prognostic for a range of important outcomes in patients undergoing systemic anticancer treatment. Risk estimates should be used to support shared decision making.

## Introduction

Aging is associated with cancer and frailty. With an aging population, the number of people aged 65 years or older diagnosed with cancer worldwide annually will double from 10.6 million in 2022 to 19.3 million by 2045.^1^ Half of older people with cancer,^2^ and some younger patients, have some degree of frailty, which is characterized by loss of biological reserves and resulting vulnerability to physical stressors. Cancer treatments can be one such stressor, and those with frailty are at increased risk of toxicity and death during cancer treatment.^2^

Eastern Cooperative Oncology Group Performance Status is widely used to determine suitability for systemic anticancer therapy, but there is a growing recognition of its limitations.^3-5^ It is increasingly recommended that frailty should be assessed and considered in decision making in patients undergoing systemic anticancer treatment.^6^^,^^7^ A number of frailty assessment tools have been tested in cancer settings; international guidance^6^^,^^7^ recommends the Geriatric-8 (G8)^8^ and Vulnerable Elders Survey-13 (VES13)^9^ for frailty screening because of their brevity and the data supporting their diagnostic and prognostic validity in cancer populations.

Prognostic factors are associated with the risk of future health outcomes in patients with a particular condition,^10^ and frailty has been widely studied as a prognostic factor in older adults with cancer.^2^ Prognostic factor studies usually employ observational designs and may be of variable methodological quality, and systematic reviews and meta-analyses are valuable to summarize the evidence.^10^ A systematic review by van Walree et al.^11^ narratively synthesized the prognostic value of the G8 frailty screening tool in older patients with cancer. Although this review found frailty scores were associated with survival and treatment-related complications, no pooled analysis was undertaken. Currently, G8 is the only frailty screening tool whose prognostic capacity in cancer patients has been systematically synthesized. No systematic reviews have explored the prognostic value of other frailty assessment tools for patients undergoing systemic anticancer treatment, and no meta-analyses have been published.^12^

This review and meta-analysis summarizes existing evidence evaluating the association between baseline frailty assessment tools and systemic anticancer treatment outcomes in adults with cancer to establish how frailty impacts the course of treatment.

## Methods

### Design and study selection (eligibility) criteria

We undertook a systematic literature review of existing prognostic factor studies reporting associations between baseline frailty assessment tools and systemic anticancer treatment outcomes. The protocol was designed using the Preferred Reporting Items for Systematic Reviews and Meta-Analyses (PRISMA) Protocol (PRISMA-P) checklist^13^ and registered on PROSPERO,^14^ and reporting aligns to the PRISMA checklist (2020).^15^

Longitudinal observational studies (cohort and case-control design) reporting on the association between validated frailty assessment tools (undertaken prior to the first cycle of systemic anticancer treatment) and important outcomes of interest (overall survival, toxicity, treatment tolerance, quality of life and functioning, and hospitalization, measured after first treatment) in adults with solid-organ malignancy undergoing, or being considered for, systemic anticancer treatment were considered for inclusion. Longitudinal data collected during clinical trials were considered for inclusion but review articles, protocols, case reports, and case series were excluded. Detailed inclusion and exclusion criteria are outlined in Methods S1.

### Frailty measures and categorization

Frailty measures and assessment tools considered eligible for inclusion in this review are outlined in Methods S1. Notably, as this review focused on the prognostic value of frailty, studies reporting on the prognostic value of geriatric assessment or individual geriatric assessment domains without providing a validated frailty measure and definition were excluded. In our synthesis, frailty was categorized in line with definitions and/or cut-points used in the included studies. For consistency, the least and most frail groups are referred to as “not frail” and “frail,” respectively.

### Information sources and search strategy

Eligible studies were identified through electronic databases (Embase, Medline, Scopus, Cumulative Index to Nursing and Allied Health Literature [CINAHL], and Cochrane Library) and supplemented through screening reference lists of included studies. The search strategy was developed with the support of an information specialist and combined terms for the key concepts of “cancer,” “frailty,” “prognostic factors,” and “SACT” (systemic anticancer treatment), using a Boolean approach (Methods S2). Searches were undertaken from database inception to January 2023. There were no limits applied to searches regarding language or year.

### Selection process

Rayyan^16^ was used to manage study selection. After removal of duplicates, 2 independent reviewers (JP, SM) screened titles and abstracts then full texts and resolved conflicts via consensus.

## Data collection process

Data on study design, participants, frailty assessments and outcomes evaluated, and results (Methods S3) were extracted from included studies independently by 2 reviewers for 25% of studies. As there was a sufficiently low level of inconsistencies (predefined as ≤1 of 10 studies with inconsistency in point estimates extracted), data from remaining studies were extracted by a single reviewer (JP, SH, JK, TN). All extracted outcome data were checked by the lead author (JP) before inclusion in the analysis. Where required, data were obtained or clarified via direct email contact with the corresponding author.

### Data items and outcome definitions

The outcomes for evaluation were predetermined (Methods S1). The main outcome of interest was overall survival, defined from baseline (diagnosis, frailty assessment, or first cycle of systemic anticancer treatment) to death from any cause. Other adverse outcomes of interest were toxicity (Common Terminology Criteria for Adverse Events graded; grade 3 or higher and/or any grade), treatment intolerance (proxy measures: treatment discontinuation, modification or delay after first cycle), deterioration in health-related quality of life ([HRQOL] assessed using a validated generic or disease-specific tool), functional decline (any measure, including decline in patient or clinician-reported functioning, or new loss of independent functioning and/or requirement for care, eg, home care or move to residential or nursing home), and unplanned hospitalization (unplanned nonelective attendance and/or admission for any reason). All available data on the association between eligible frailty assessments and outcomes of interest were extracted. Where multiple follow-up timepoints were reported, the longest follow-up duration available was extracted. Full details of data extracted from included studies are summarized in Methods S3.

### Study risk of bias assessment

Risk of bias was assessed using the Quality of Prognosis Studies in Systematic Reviews tool.^17^ Two independent reviewers (JP, EK) assessed 20% of studies; as there were no discrepancies in the overall risk of bias assessment, the remaining studies were assessed by a single reviewer (EK).

### Effect measures, synthesis methods, and statistical analysis

A summary of included studies and their reported associations between eligible frailty assessment tools and outcomes were tabulated and summarized using appropriate summary statistics. Findings from some studies were reported in more than 1 article. Study characteristics, frailty assessments evaluated, and risk of bias are summarized for all eligible articles. To avoid multiple reports from a single study biasing the findings of our synthesis, data on the association between frailty and outcomes were only synthesized once per study (taking outcome data from the article and/or report with the largest sample size and/or follow-up period). Full details on sources and handling of multiple reports are outlined in Methods S4.

Decision rules^18^ were applied to systematically report the best available summary statistics from included studies and to minimize reporting bias.^19^^,^^20^ Outcomes were reported using hazard ratios (HR) for death and odds ratio (OR) for other adverse outcomes (toxicity, treatment intolerance, HRQOL, functional decline, hospitalization) in not frail (fit) vs frail patients, with 95% confidence intervals (CIs), where available. Adjusted ratios (hazard ratios or odds ratios) were prioritized, with unadjusted ratios (calculated from raw data if required) included if adjusted data were unavailable. Full details of decision rules are described in Methods S5.

We undertook meta-analyses to synthesize hazard ratios for mortality and odds ratios for other adverse outcomes,^21^ presenting 1 forest plot per outcome measure, with each binary frailty assessment tool reported as a subgroup. Ordinal frailty assessments were not synthesized in meta-analysis because of heterogeneity in how frailty categories were combined and/or compared but are reported narratively in tabulated form. Forest plots^22^ were generated via generic inverse variance random effects modeling^23^ using RevMan v5.3. Pooled estimates for hazard ratio and odds ratio are provided by frailty tool subgroup and overall, with 95% confidence intervals and between-study heterogeneity (*I*^2^).^23-25^ Individual studies contributing to the meta-analysis were labeled stating whether the estimate reported was adjusted or unadjusted.

The results from multiple frailty assessment tools were included in some of the studies. In the primary analysis, data on all eligible frailty tools were included to provide robust estimates for the association between each frailty tool subgroup and outcome. However, to avoid multiple reports from a single study biasing the overall pooled estimate for the association between overall frailty and outcome, a secondary analysis was undertaken where only 1 point estimate from each study was included (Methods S5). Sensitivity analyses^26^ were undertaken to evaluate sources of statistical heterogeneity and the impact of bias on pooled estimates, relating to study characteristics (frailty tool, adjustment for covariates,^20^ and study quality) and patient characteristics (cancer site and disease stage and treatment intent). We investigated publication bias within meta-analyzed studies using funnel plots.^23^ No assessment of the certainty in the body of evidence for each outcome was undertaken, because of the large number of potential frailty assessment/outcome dyads and the lack of established methodology within prognosis factor research.^10^

## Results

### Study selection

**Figure 1.**
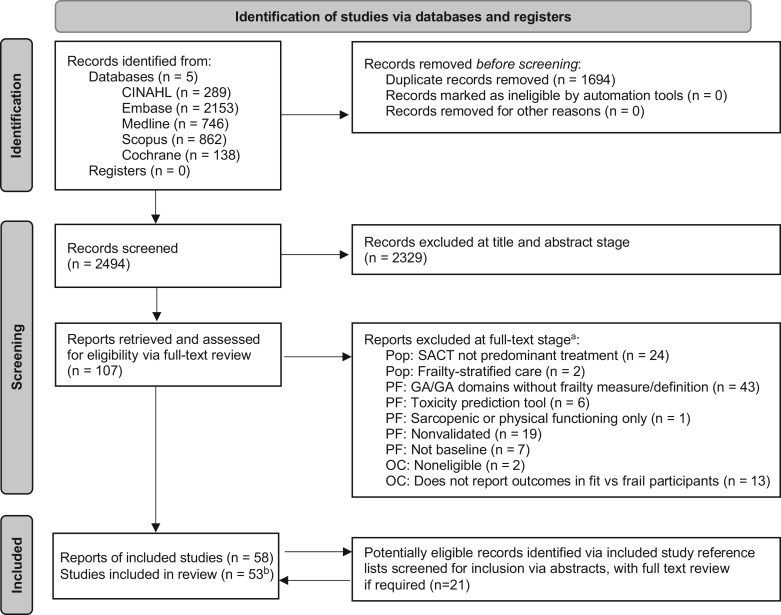
Preferred Reporting Items for Systematic Reviews and Meta-Analyses diagram. ^a^Exclusion reasons relate to the study population, prognostic factor, and outcome. More than 1 exclusion reason is listed for some studies.^ b^Of 53 included studies, 52 were identified via the initial searches and 1 was identified from reference lists. Adapted from The 2020 PRISMA statement.^15^ Abbreviations: GA = geriatric assessment; OC = outcome; PF = prognostic factor; Pop = population; SACT = systemic anticancer treatment.

After deduplication, 2494 citations were identified. A total of 58 articles reporting on data from 53 studies were included. The review process is summarized in a PRISMA flow diagram (Figure 1). The most common reason for an article to be excluded at full text stage (n = 43 of 107 excluded after full text review) was that it reported on geriatric assessment and/or geriatric assessment domains as a prognostic factor without providing a frailty measure and definition.

### Study characteristics

Characteristics of the 58 included articles are outlined in Table 1. The majority (56 of 58) were published in the last decade (since 2013). All but 4 articles focused primarily on older patients; the most used age cutoff was 70 years and older. The average age of patients ranged between 65 and 79.2 years, with the youngest and oldest patients included across all articles aged 30 and 97 years, respectively. Study populations were heterogenous with 39% of articles (n = 23) including patients with a mix of different cancers. Of the articles focusing on a specific tumor site, the most studied sites were gastrointestinal ([GI] n = 15, 25%; upper GI: n = 8; lower GI: n = 5; mixed upper and lower, n = 2), breast (n = 6), prostate (n = 6), and lung (n = 5). Most articles focused on patients with advanced cancers and/or those being treated with palliative intent (n = 35, 60%). Two (3%) focused on those with early disease stage and/or curative treatment intent, and the remaining 21 (47%) included a mix of disease stages and/or treatment intents.

**Table 1.** Summary of included studies (study characteristics, included patients, frailty assessments and outcomes evaluated and overall risk of bias).

Study authors, year	Cohort design	Tumor site	Age, y	Disease stage, treatment intent^a^	SACT type^b^	No.^b^	Age in years^c^	ECOG PS 0-1 %	Frailty measure(s), categorization, and % by category^b^	Survival	Toxicity	Treatment tolerance	Functioning	HR QOL	Hospitalisation	RoB
Aaldriks^84^^,^^d^, 2011^e^	Prospective	Various	70 and older	Stage NES, mixed intent (55% palliative)	Chemotherapy	202	77.2 (71-92)	NR	GFI: <4, 63%; ≥4, 37%	Y	N	Y	N	N	N	Mod
Aaldriks^83^^,^^d^, 2013^e^	Prospective	Breast	70 and older	Advanced, intent NES	Various (chemotherapy and/or traztuzumab)	55	76 (4.8)	71%	GFI: <4, 49%; ≥4, 51%	Y	N	Y	N	N	N	Mod
Aaldriks^85^^,^^d^, 2013^e^	Prospective	Colorectal	70 and older	Mixed stage and/or intent (62% metastatic/palliative)	Chemotherapy	143	75 (70-92)	87%	GFI: <4, NR; ≥4, 24%	Y	N	Y	N	N	N	Mod
Aaldriks^82^^,^^e^, 2016^e^	Prospective	Various	70 and older	Stage NES, mixed intent (58% palliative)	Chemotherapy	494	75 (70-92)	NR	GFI: <4, 70%; ≥4, 30%	Y	N	Y	N	N	N	Mod
Akbıyık^33^, 2022	Prospective	Various	65 and older	Mixed stage and/or intent (50% palliative)	Chemotherapy	44 (40 alive and on treatment)	70 (66-83)	84%	Edmonton: <7, 73%; ≥7, 27%	N	Y	N	N	N	N	Mod
Alibhai^69^, 2021	Prospective	Prostate	65 and older	Advanced, intent NES	Chemotherapy or NAAT (abiraterone or enzalutamide)	175 (71 chemo and 104 NAAT)	Chemo 73.6 (65-90); NAAT 75.9 (65-97)	NR	G8: >14, NR; ≤14 73/50% VES13: <3, NR; ≥3 46/29% (chemo/NAAT patients, respectively)	N	Y	N	N	N	N	Mod
Banna^58^, 2022	Prospective	Prostate	70 and older	Advanced, intent NES	NAAT (enzalutamide)	234	78 (IQR = 73-82)	NR	G8: >14, 62%; ≤14, 38%	Y	N	N	N	N	N	Mod
Basso^50^, 2008	Retrospective	Various	70 and older	Mixed stage and/or intent (80% palliative)	Chemotherapy	117	75 (70-92)	NR	GA-based: fit 33%, vulnerable 33%, frail 34%	Y	Y	N	N	N	N	Mod
Bauman^96^, 2012	Prospective	Non-small cell lung cancer	70 and older	Advanced, intent NES	Combination (chemotherapy and tyrosine kinase inhibitor)	34	74.5 (70-86)	82%	VES13: <3, NR; ≥3, 38%	Y	N	N	N	N	N	High
Beardo^59^, 2019	Retrospective	Prostate	75 and older	Advanced, palliative	NAAT (abiraterone or enzalutamide)	134 (70 aged 75 years and older)	NR	96%	G8: >14, NR; ≤14, NR	Y	N	N	N	N	N	Mod
Bruijnen^71^, 2022	Prospective	Melanoma	70 and older	Advanced, intent NES	Immunotherapy	92	76 (70‐89)	87%	G8: >14, 71%; ≤14, 29%	N	Y	N	N	N	Y	Mod
Brunello^51^, 2013	Retrospective	Renal cell carcinoma	70 and older	Advanced, intent NES	Targeted therapy (sunitinib)	68 (34 with GA undertaken)	74 (70-88)	87%	GA-based: fit 38%, vulnerable 41%, frail 21%	Y	Y	N	N	N	N	High
Cavdar^70^, 2022	Prospective	Various	65 and older	Majority advanced (95% stage 3 or 4), mixed intent (% NES)	Chemotherapy	208	70 (65-86)	73%	G8: >14, 25%; ≤14, 75% VES13: <3, 59%; ≥3, 48%	N	Y	Y	N	N	Y	Low
Chakiba^79^, 2019	Prospective	Various	70 and older	Mixed stage (51% advanced), intent NES	Chemotherapy	292 (287 with data for fxn decline)	77 (70-93)	77%	G8: >14, 18%; ≤14, 82%	N	N	N	Y	N	N	Low
Chan^72^, 2021	Prospective	Various	65 and older	Mixed stage (94% stage 3 or 4) and intent (64% palliative)	Various (chemotherapy 81%, targeted therapy alone 19%)	259	73.4; unit unclear (65-93)	89%	G8: >14, 27%; ≤14, 73%	N	Y	Y	N	N	Y	Low
Chiusole^52^, 2023	Retrospective	Sarcoma	70 and older	Advanced, intent NES	Chemotherapy (75%, rest unspecified)	101	77 (70-91)	75%	GA-based: fit 39%, vulnerable 46%, frail 16%	Y	Y	N	N	N	N	Mod
Decoster^39^, 2017	Prospective	Colorectal	70 and older	Mixed stage (80% stage 3 or 4) and treatment intent	Various (chemotherapy 57%, surgery 45%, radiotherapy 7%)	193 (164 and 109 with functioning and toxicicity data, respectively)	77 (70-89)	75%	G8: >14, 25%; ≤14, 75% fTRST: 0, 19%; ≥1, 81%	N	Y	N	Y	N	N	Mod
Decoster^38^, 2017	Prospective	Lung	70 and older	Mixed stages (87% stage 3 or 4) and treatment intents	Various (chemotherapy 51%, radiotherapy, 43%, surgery 8%, best supportive care 17%)	245	76 (70-93)	66%	G8: >14, 17%; ≤14, 83% fTRST: 0, 9%; ≥1, 91%	Y	N	N	Y	N	N	Low
Feliu^99^^,^^e^, 2020	Prospective	Various	70 and older	Mixed stage (58% stage 4) and treatment intent (65% palliative)	Chemotherapy (27% with concurrent radiotherapy)	540	77 (70-92)	90%	VES13: <3, 50%; ≥3, 50%	N	Y	N	N	N	N	Mod
Feliu^97^^,^^e^, 2021	Prospective	Various	70 and older	Mixed (54% stage 4), intent NES	Chemotherapy	342	78 (70-92)	87%	VES13: <3, 49%; ≥3, 51%	Y	N	N	N	N	N	Mod
Ferrero^101^, 2018	Prospective	Gynecological malignancies	70 and older	Mixed stage, intent NES	Chemotherapy	84	75 (70-89)	NR	VES13: <3, 57%; ≥3, 43%	N	N	Y	N	N	N	Mod
Francolini^78^, 2023	Prospective	Prostate	70 and older	Advanced, intent NES	Chemotherapy	118 (93 in docetaxel subgroup)	75 (IQR = 72-78)	NR	G8: >14, 29%; ≤14, 71% (docetaxel subgroup)	N	N	Y	N	N	N	Mod
Gebbia^60^, 2021	Retrospective	Pancreatic	70 and older	Advanced, intent NES	Chemotherapy	40	74 (70-81)	85%	G8: >14, 15%; ≤14, 85%	Y	Y	Y	N	N	N	Mod
Gironés^53^, 2018	Prospective	Non-small cell lung cancer	70 and older	Advanced, intent NES	Chemotherapy	93	77 (IQR = 70-92)	83%	GA-based: fit 42%, vulnerable 33%, frail 25%	Y	Y	N	N	N	N	Mod
Hamacher^61^, 2023	Retrospective	Soft tissue sarcoma	60 and older	Advanced, intent NES	Targeted therapy or chemotherapy	118	71 (60-88)	93%	G8: >14, 48%; ≤14, NR	Y	Y	Y	N	N	N	Mod
Hamaker^86^, 2014	Prospective	Breast	65 and older	Advanced, palliative	Chemotherapy	73	75.5 (65.8-86.8)	78%	GFI: <4, NR; ≥4, 56%	Y	Y	N	N	N	N	Mod
Hay^47^, 2019	Prospective	Gynecological	65 and older	Stage NES, treatment curative (66% adjuvant, 33% neoadjuvant)	Chemotherapy (71% also received surgery)	80	72 (65-87)	NR	FRAIL: 0-1, 60%; 2-3, 28%; 4-5, 13%	N	N	Y	N	N	Y	Mod
Jespersen^62^, 2021	Prospective	GI	70 and older	Advanced, intent NES	Chemotherapy	170	75.5 (70-88)	79%	G8: >14, NR; ≤14, 74% VES13: <3, NR; ≥3, 29%	Y	N	N	Y	N	N	Mod
Kenis^40^, 2017	Prospective	Various	70 and older	Mixed stage and/or intent (>60% advanced/palliative)	Chemotherapy	439 (387 with fxn data)	75 (70-95)	75%	G8: >14, 25%; ≤14, 75% fTRST: 0, 19%; ≥1, 81%	N	N	N	Y	N	N	Low
Kirkhus^54^, 2017	Prospective	Various	70 and older	Mixed stage, palliative	Various (chemotherapy and/or other SACT; 7% received nonsystemic palliative treatment)	288	77 (70-95)	85%	GA-based: nonfrail 51%, frail (vulnerable and frail) 49%	Y	N	N	N	N	N	Mod
Kobayashi^63^, 2022	Prospective	Pancreatic	76 and older	Advanced, palliative	Various (chemotherapy 81%, other 19%)	233 (183 chemotherapy patients)	NR (76-88)	91%	G8: >14, 9%; ≤14, 90%	Y	N	N	N	N	N	Mod
Kotzerke^73^, 2019	Prospective	Various	65 and older	Mixed stage (84.6% stage 3 or 4) and intent	Chemotherapy	104	72.8 (65-82)	96%	G8: >14, NR; ≤14, 74%	N	Y	N	N	N	N	Mod
Kroep^87^, 2015	Prospective	Colorectal	65 and older	Advanced, palliative	Chemotherapy	67	77 (66-88)	87%	GFI: <4, NR; ≥ 4, NR	Y	N	N	N	N	N	Low
Li^64^, 2022	Prospective	Colorectal	75 and older	Mixed stage (85% stage 3 or 4) and intent (80% palliative)	Chemotherapy +/- targeted therapy (anti-VEGF/EGFR)	28 (20 with complete data)	78.5 (75-88)	75%	G8: >14, 45%; ≤14, 55%	Y	N	N	N	N	N	Low
Luciani^100^, 2015	Retrospective	Various	66 and older	Mixed stage (70.4% metastatic) and intent (32% adjuvant and/or neoadjuvant)	Chemotherapy	648	76.2 (66-90)	89%	VES13: <3, 56%; ≥3, 44%	N	Y	Y	N	N	N	Mod
Manokumar^102^, 2016	Prospective	Prostate	65 and older	Advanced, intent NES	Chemotherapy	29	77.3 (4.5)	NR	VES13: <3, 38%; ≥3, 62%	N	N	N	Y	Y	N	Mod
Mathur^93^, 2022	Retrospective	Non-small cell lung cancer	NR	Advanced, intent NES	Chemotherapy (81% also radiotherapy)	426	65 (30-85)	81%	mFI: 0, 41%; 1-2, 46%; ≥3, 13%	Y	N	Y	N	N	Y	Mod
Moth^31^, 2020	Prospective	Various	65 and older	Advanced, palliative	Chemotherapy	102	74 (65-86)	81%	CFS (CSHA): 0-3, 65%; 4-7, 35%	Y	N	N	N	N	N	Mod
Nakazawa^74^, 2021	Prospective	GI	70 and older	Advanced, palliative	Chemotherapy	93	76 (70-88)	91%	G8: >14, 18%; ≤14, 82%	N	Y	N	N	N	N	Mod
Orum^55^, 2018	Retrospective	Various	70 and older	Mixed stage (41% metastatic disease) and intent (palliative 53%)	Chemotherapy (initial treatment modality in 66% vs radiotherapy 44%)	217	75 (70-88)	NR	GA-based: fit, 13%; vulnerable, 35%; frail, 52%	Y	N	Y	N	N	Y	High
Pearce^65^, 2022	Retrospective	Gastroesophageal	None	Advanced, palliative	Chemotherapy	514 (500 with G8 results)	76 (51-96)	68%	G8: >14, 8%; ≤14, 89%	Y	N	N	N	N	N	Low
Phaibulvatanapong^45^, 2018	Prospective	Various	70 and older	Stage NES, mixed intent (palliative 66%)	Chemotherapy (87%)	151 (132 with fxn data)	75.37 (70.0-88.1); units unclear	89%	Fried: <3, NR; ≥3, 29%	N	Y	Y	N	Y	N	Mod
Procaccio^56^, 2022	Prospective	Colorectal	70 and older	Advanced, intent NES	Chemotherapy (25% did not receive)	488	76.1	84%	GA-based: fit, 52%; vulnerable, 28%; frail, 20%	Y	N	N	N	N	N	High
Ramsdale^98^, 2013	Prospective	Colorectal	65 and older	Advanced, intent NES	Chemotherapy	38	72 (65-89)	91%	VES13: <3, NR; ≥3, NR	Y	N	N	N	N	N	High
Rier^57^, 2022	Prospective	Various	65 and older	Stage NES, mixed intent (in solid organ patients: 40% palliative)	Chemotherapy	291 (121 with solid organ malignancy)	72 (IQR = 68-77)	91%	G8: >14, NR; ≤14, 51/57% GA-based: fit, 14/22%; vulnerable, 14/53%; frail, 37/25% (solid organ; palliative/neoadjuvant)	Y	N	Y	N	N	N	Mod
Rittberg^94^, 2021	Retrospective	Pancreatic	65 and older	Advanced, palliative	Chemotherapy	87	71 (65-88)	79%	mFI: 0, 23%; 1, 32%; 2, 29%; ≥3, 16%	Y	N	Y	N	N	Y	High
Ruiz^44^, 2019	Prospective	Non-small cell lung cancer	18 and older	Advanced, palliative	Chemotherapy	48	69 (42-86)	NR	Fried: <3, 73%; ≥3, 27%	Y	Y	Y	N	N	Y	Mod
Runzer-Colmenares^75^^,^^d^, 2019^e^	Retrospective	Prostate	NR	NES	Chemotherapy	161	79.2 (4.3)	NR	G8: >14, 76%; ≤14, 24% VES13: <3, 73%; ≥3, 26%	N	Y	N	N	N	N	Unable to assess
Runzer-Colmenares^46^^,^^e^, 2020	Retrospective	Various	60 and older	NES	Chemotherapy	496	79.2 (4.3)	NR	G8: >14, 77%; ≤14, 23% VES13: <3, 80%; ≥3 20% Fried: <3, 84%; ≥3, 26%	N	Y	N	N	N	N	Mod
Sakamoto^37^, 2021	Prospective	Colorectal	65 and older	Advanced, intent NES	Chemotherapy	30	73 (65-81)	89%	G8: >14, 27%; ≤14, 73% fTRST: 0-1, 60%; 2-5, 40%	Y	N	N	N	N	N	Low
Shachar^95^, 2022	Prospective	Various	65 and older	Advanced, intent NES	Various (chemotherapy (+/- biologic) or immunotherapy)	48	72 (IQR = 68-76)	NR	FI (CFI): 0-0.2, 42%; 0.2-0.35, 27%; >0.35, 31%	N	Y	N	N	N	N	Mod
Shah^30^, 2022	Retrospective	GI	None	Advanced, palliative	Various (SACT 62%, best supportive care 35%, surveillance 4%)	200	70.4; units unclear	57%	CFS: 1-3, 62%; 4-5, 26%; 6-9, 13%	Y	N	N	N	N	N	Mod
Von Minckwitz^76^, 2015	Prospective	Breast	65 and older	Early, curative	Chemotherapy	391	72 (65-84)	100%	G8: >14, 83%; ≤14, 17%	N	Y	Y	N	N	N	Mod
Weiss^92^, 2020	Prospective	Non-small cell lung cancer	70 and older	Advanced, intent NES	Chemotherapy	42	76.3 (71-84)	79%	FI (FI-CSGA): <0.2, 19%; 0.2-0.35, 39%; >0.35, 42%	Y	Y	N	N	N	N	Mod
Wildiers^66^^,^^e^, 2022	Prospective	Breast	60 and older	Advanced, intent NES	HER2 therapy +/- chemotherapy	80	76.2/77.3 (61.4-91.4); units unclear	77%	G8: >14, 30%; ≤14, 69%	Y	N	N	N	N	N	High
Wildiers^67^^,^^d^, 2018^e^	Prospective	Breast	60 and older	Advanced, palliative	HER2 therapy +/- chemotherapy	80	76.2/77.3 (61.4-91.4); units unclear	77%	G8: >14, 30%; ≤14, 69%	Y	N	N	N	N	N	Low
Winther^77^, 2019	Prospective	Colorectal	70 and older	Advanced, intent NES	Chemotherapy	160	78 (75-81)	80%	G8: >14, 28%; ≤14, 69% VES13: <3, 71%; ≥3, 23%	N	Y	Y	N	N	Y	Low
Wu^68^, 2023	Prospective	Thoracic (lung and mesothelioma)	70 and older	Advanced, palliative	Various (chemotherapy, targeted therapy, or immunotherapy)	201 (127 receiving chemotherapy)	77 (70-90)	68%	G8: >14, 26%; ≤14, 75%	Y	Y	Y	N	N	Y	Mod

Abbreviations: +/- = with or without; CFI = Carolina frailty index; CFS = clinical frailty scale; chemo = chemotherapy; CI = confidence interval; CSGA = cancer-specific geriatric assessment; CSHA = Canadian Study of Health and Aging; EGFR = epidermal growth factor receptor; ECOG = Eastern Cooperative Oncology Group; fTRST = Flemish version of the Triage Risk Screening Tool; G8 = geriatric-8; GA = geriatric assessment (Balducci-based); GFI = Groningen Frailty Indicator; GI = gastrointestinal; IQR = interquartile range; mFI = modified Frailty Index; mod = moderate; N = no; NAAT = novel anti-androgen therapy; NES = not explicitly stated; NR = not reported; PS = performance status; SACT = systemic anticancer therapy; VEGF = vascular endothelial growth factor; VES13 = vulnerable elders survey-13; Y = yes.

a Percentages are rounded to nearest integer (totals may not equal 100% because of rounding or missing data).

b No. relates to number of participants in the main analysis (n in extracted frailty and outcome analysis, if different). Participant characteristics relate to main analysis unless specified.

c Age is reported as median (range) unless specified.

d Do not directly contribute to the tabulated results of this review.

e Indicates articles that are duplicate reports of the same study.

### Risk of bias of included studies

Quality of Prognosis Studies in Systematic Reviews assessments evaluated study quality. In terms of the overall risk of bias rating, most articles (n = 39, 67%) were deemed to have moderate overall risk of bias, with 11 (19%) and 7 (12%) being deemed to have low and high overall risk, respectively. Table 1 summarizes overall risk of bias; Table S1 provides a breakdown of the risk within each of the 6 domains assessed.

### Frailty assessment tools and outcomes evaluated

Table 2 provides an overview of the 9 frailty assessment tools applied (Clinical Frailty Scale [CFS], Edmonton, Flemish Triage Risk Screening Tool [fTRST], Fried, GA-based, Groningen Frailty Indicator (GFI), G8, frailty indices, and VES13) and the articles reporting their association with each outcome. Most frailty assessment tools reported frailty as a binary measure, although 3 tools (GA-based, frailty indices, and the Fried-based FRAIL scale) grouped patients into 3 or more ordinal frailty categories.

**Table 2.** Overview of frailty assessment tools used in included studies

Frailty assessment tool^a^	Frailty score range and definition^b^	Outcome reported: No. of articles
**CFS^27^** 9-point ordinal scale for assessing global fitness and frailty based on the single domain of functioning.^27^^,^^28^ It was adapted from the Canadian Study on Health and Aging 7-point ordinal scale.^29^ Scores range from 1 “very fit” to 7 “severely frail” or 9 “terminally ill” (CHSA and CFS, respectively).	Range: 1-7/9 Definition: varies, but most studies define not frail <4, prefrail (aka vulnerable) ≥4	Any outcome: 2 Survival: 2 (CFS^30^ and CSHA^31^) Toxicity: 0 Treatment tolerance: 0 Functioning: 0 Health-related QOL: 0 Hospitalization: 0
**Edmonton Frail Scale^32^** 9 item structured assessment tool Domains assessed: general health status, functional independence, social support, medication use, nutrition, mood, continence, cognition performance (Clock test), functional performance (Timed Get Up & Go)	Range: 0-17 Definition: not frail <7, frail ≥ 7	Any outcome: 1 Survival: 0 Toxicity: 1^33^ Treatment tolerance: 0 Functioning: 0 Health-related QOL: 0 Hospitalization: 0
**Flemish version—Triage Risk Screening Tool^34^** 5-item structured tool Domains assessed: cognition, independence at home and mobility, recent hospitalization, polypharmacy	Range: 0-6 Definition: varies Geriatric population:^35^ not frail 0-1, frail (aka abnormal or geriatric risk profile) ≥2 Cancer populations:^36^ not frail 0, frail ≥1	Any outcome: 4 Survival: 2^37^^,^^38^ Toxicity: 1^39^ Treatment tolerance: 0 Functioning: 3^38-40^ Health-related QOL: 0 Hospitalization: 0
**Fried Frailty Score (aka index or indicator)^41^** Presence/absence of phenotypic features: 1. Shrinking: unintentional weight loss 2. Weakness: grip strength 3. Poor endurance and energy: self-reported exhaustion 4. Slowness: walking speed 5. Low physical activity level: self-reported activity (kilocalories per week) FRAIL scale^42^^,^^43^ is a patient self-report questionnaire based on the Fried Frailty Score	Range: 0-5 Definition: varies Fried frailty score: not frail <3, frail ≥3 FRAIL scale: not frail (aka fit or robust) 0-1, intermediate frailty 2-3, frail 4-5	Any outcome: 3 (Fried) + 1 (FRAIL) Survival: 1 (Fried^44^) Toxicity: 3 (Fried^44-46^) Treatment tolerance: 2 (Fried^44^^,^^45^) and 1 (FRAIL^47^) Functioning: 0 Health-related QOL: 1 (Fried^45^) Hospitalization: 1 (Fried^44^) + 1 (FRAIL^47^)
**GA–based frailty tool ^48^** GA consisting of multiple domains. In oncology, the Balducci criteria^48^ have been applied (measured by different tools), based on earlier work by Hamerman in general populations.^49^ Domains include functioning, comorbidities, cognition and mood, geriatric syndromes Balducci criteria categorizes stage of aging (primary, intermediate, and secondary or frailty) and is adapted to fit, vulnerable, or frail in most studies.	Range: N/A (see below) Definition: Not frail (aka fit): no functional dependence, comorbidities, or geriatric syndromes Intermediate (aka vulnerable): dependence in ≥1 ADL, significant but not life-threatening comorbidity, may have mild memory disorder or depression Frail: dependence in ≥1 ADL, ≥1 geriatric syndromes, significant comorbidity (≥3 conditions and/or significant influence on daily function)	Any outcome: 8 Survival: 8^50-57^ Toxicity: 4^50-53^ Treatment tolerance: 2^55^^,^^57^ Functioning: 0 Health-related QOL: 0 Hospitalization: 1^55^
**Geriatric-8^8^** 8-item structured questionnaire. Domains assessed: nutrition, weight loss, mobility, cognition and depression, polypharmacy, age, self-rated health	Range: 0-17 Definition: not frail (aka fit or normal) >14, frail (aka vulnerable or abnormal) ≤14	Any outcome: 28 Survival: 14^37^^,^^38^^,^^57-68^ Toxicity: 14^39^^,^^46^^,^^60^^,^^61^^,^^68-77^ Treatment tolerance: 9^57^^,^^60^^,^^61^^,^^68^^,^^70^^,^^72^^,^^76-78^ Functioning: 5^38-40^^,^^62^^,^^79^ Health-related QOL: 0 Hospitalization: 4^68^^,^^70-72^
**Groningen Frailty Indicator^80^** 15-item structured questionnaire. Domains assessed: physical, cognitive, social, psychological	Range: 0-15 Definition^81^: not frail <3, frail ≥4	Any outcome: 6 Survival: 6^82-87^ Toxicity: 2^69^^,^^86^ Treatment tolerance: 4^82-85^ Functioning: 0 Health-related QOL: 0 Hospitalization: 0
**Frailty indices: cumulative deficits methods** Frailty defined as an accumulation of deficits^88^ following Searle et al.’s^89^ method. A total of 30-40 variables covering a range of body systems scored 0-1 (none to full deficit). GA domains and/or measures may contribute.^90^ Individual deficit scores summated into total Frailty Index score. mFI assesses accumulated deficits within 11 variables available in the National Surgical Quality Improvement Program database; it does not follow the Searle method but is widely validated in cancer surgical settings.^91^	Range: see below Definition: variable. FI: range = 0-1. Not frail (aka fit) ≤0.2, prefrail 0.20-0.35, frail >0.35 mFI: range = 0-11 deficits. Not frail 0, mild-moderate frailty 1-2, frail/severe frailty ≥3	Any outcome: 4 (2 mFI, 1 FI-CSGA, 1 CFI) Survival: 3 (FI-CSGA^92^ and mFI ^93^^,^^94^) Toxicity: 2 (CFI^95^ and FI-CSGA^92^) Treatment tolerance: 2 (mFI^93^^,^^94^) Functioning: 0 Health-related QOL: 0 Hospitalization: 2 (mFI^93^^,^^94^)
**Vulnerable Elders Survey-13^9^** 13-item structured questionnaire. Domains assessed: age, self-rated health, limitations in physical activities and functional disabilities of daily living (ADL and instrumental ADL)	Range: 0-10 Definition: not frail <3, frail ≥3	Any outcome: 13 Survival: 4^62^^,^^96-98^ Toxicity: 7^46^^,^^69^^,^^70^^,^^75^^,^^77^^,^^99^^,^^100^ Treatment tolerance: 5^62^^,^^70^^,^^77^^,^^100^^,^^101^ Functioning: 1^102^ Health-related QOL: 1^102^ Hospitalization: 2^70^^,^^77^

Abbreviations: ADL = activities of daily living; aka = also known as; CFI = Carolina frailty index; CFS = clinical frailty scale; CSGA = cancer-specific geriatric assessment; CSHA = Canadian Study of Health and Aging; FI = frailty index; GA = geriatric assessment; mFI = modified FI; N/A = not applicable; QOL = quality of life.

a Reference provided for each frailty tool is the primary reference.

b Definitions reported reflect those validated cut-points adopted in eligible studies; other cut-points may be applied in the wider literature.

Table 3 summarizes the associations between frailty assessments and outcomes and the number of participants, follow-up durations, and covariates in adjusted models. Across all studies, the number of participants included in frailty and outcome analyses ranged from 20 to 648, and frailty and outcome associations are reported for a total of 9505 patients. The frailty assessment tools most commonly used were the G8 (n = 28 articles), VES13 (n = 13), and GA-based (n = 8) tools. The outcomes most commonly evaluated were survival (n = 36), toxicity (n = 26, of which 25 reported on grade 3 or higher toxicity, 1 reported on grade 2 or higher toxicity only), treatment tolerance (n = 21, of which 19 reported on treatment cessation), and hospitalization (n = 10). Six articles evaluated functional decline, 5 of which used patient-reported outcomes based on activities of daily living and 1 of which used clinician assessment via performance status. Two articles evaluated HRQOL. Adjustment for covariates was undertaken in 36% (40 of 112) of the reported frailty and outcome analyses across all studies.

**Table 3.** Summary of study results relating to the association between frailty assessment tools and outcomes

Study author, year	No.	Outcome,^a^ follow-up time point^b^	Between group comparison^c^	Covariates in adjusted models
**Clinical frailty scale (not frail 1-3, intermediate or frail ≥4)**				
Shah^30^, 2022	200	Mortality, not stated	Adjusted HR = 1.5 (95% CI = 1.2 to 1.9)	Age, gender, primary site, PS
Moth^31^, 2020 (CHSA)	102	Mortality, median = 19 (0-27) months	Adjusted HR = 4.16 (95% CI = 2.34 to 7.40)	Estimated expected survival time, PS, cancer type, hemoglobin, self-rated health, IADL, weight loss, cancer and aging research group toxicity score, GA score
**Edmonton (not frail <7, frail ≥7)**				
Akbıyık^33^, 2022	40	Toxicity, up to 2 months	Unadjusted OR^d^ = 0.39 (95% CI = 0.10 to 1.56)	N/A
**FI (CFI/CSGA: not frail <0.35, frail >0.35, mFI: grouping and/or labeling varies)**				
Shachar^95^, 2022 (CFI)	48	Toxicity, not stated	Unadjusted OR^d^ = 0.78 (95% CI = 0.21 to 2.84) for grade 2 or higher CTCAE toxicity	N/A
Weiss^92^, 2020 (FI-CSGA)	42	Mortality, not stated	Median overall survival = 14.2 months (not frail) vs 7.5 months (frail); *P* =.045	N/A
	42	Toxicity, up to 6 cycles	Not statistically significant	N/A
Mathur^93^, 2022 (mFI)	426	Mortality, not stated	adj HR = 1.03 (95% CI = 0.83 to 1.29) for mFI 1-2 (intermediate frailty); adj HR = 0.73 (95% CI = 0.52 to 1.03) mFI ≥3 (frailty)	PS, age, sex, histology, mutation status, no. of metastases, presence of brain metastases, first line platinum used, smoking status
	426	Treatment intolerance (cessation), within 4 cycles	Unadjusted OR^d^ = 1.01 (95% CI = 0.65 to 1.56) for mFI 1-2 (moderate frailty); 0.73 (95% CI = 0.37 to 1.44) for mFI ≥3 (frailty)	N/A
	426	Hospitalization, within 30 days of a chemotherapy course	Not statistically significant for mFI 1-2 (moderate frailty); unadjusted *P =* .095 for mFI ≥3 (frail)	N/A
Rittberg^94^, 2021 (mFI)	87	Mortality, not stated	adj HR = 0.71 (95% CI = 0.38 to 1.35) mFI 1 (mild frailty); 0.98 (95% CI = 0.52 to 1.86) mFI 2 (moderate frailty); 0.70 (95% CI = 0.34 to 1.44) mFI ≥3 (severe frailty)	Chemotherapy used, PS
	87	Treatment intolerance (modification), not stated	Unadjusted OR^d^ = 0.54 (95% CI = 0.17 to 1.75) for 1 (mild frailty); 1.38 (95% CI = 0.39 to 4.92) for 2 (moderate frailty); 0.97 (95% CI = 0.23 to 4.04) for mFI ≥3 (severe frailty)	N/A
	87	Hospitalization (attendance), within 3 months of treatment start	Unadjusted *P =* .882	N/A
**Fried (Fried frailty score: not frail <3, frail ≥3; FRAIL scale: not frail 0-1, intermediately frail 2-3, frail 4-5)**				
Hay^47^, 2019 (FRAIL scale)	80	Treatment intolerance (cessation), within 3 cycles (neoadjuvant) or 6 cycles (adjuvant)	Unadjusted OR^d^ = 2.37 (95% CI = 0.43 to 12.81) for intermediately frail; 6.43 (95% CI = 1.08 to 38.41) for frail	N/A
	80	Hospitalization, within 3 cycles (neoadjuvant) or 6 cycles (adjuvant)	Not statistically significant	N/A
Phaibulvatanapong^45^, 2018 (Fried frailty score)	151	Toxicity, not stated	Adjusted OR = 1.16 (95% CI = 0.47 to 2.87)	Primary cancer, combined chemotherapy, line of chemotherapy, PS, body mass index, nutritional status, caregiver needed
	151	Treatment intolerance (cessation), 3 months	Unadjusted OR = 0.91 (95% CI = 0.40 to 2.09)	N/A
	132	HRQOL (continuous, mean FACT-G score difference), baseline vs 3 months or systemic anticancer therapy end	Mean FACT-G score difference = -3.91 (9.76) for not frail vs -0.42 (10.67) for frail group (MCID not referenced); *P =* .08 (univariate analysis); *P =* .21 (multivariate analysis)	PS, pretreatment weight loss, nutritional status
Ruiz^44^, 2019 (Fried frailty score)	48	Mortality, not stated	Unadjusted HR = 1.03 (95% CI = 0.51 to 2.11)	N/A
	48	Toxicity, 2 cycles	Adjusted OR = 7.03 (95% CI = 1.11 to 44.55)	Age, body surface area, comorbidity
	48	Treatment intolerance (modification), 3 cycles	Not statistically significant	N/A
	48	Hospitalization, 2 cycles	Not statistically significant	N/A
Runzer-Colmenares^46^, 2020 (Fried frailty score)	496	Toxicity, not stated	Unadjusted OR^d^ = 11.21 (95% CI = 7.02 to 17.92)	N/A
**Flemish version of the Triage Risk Screening Tool (not frail 0, frail ≥1 unless specified)**				
Decoster^39^, 2017a	109	Toxicity, not stated	Unadjusted *P =* .740 for hematological toxicity Unadjusted *P =* .831 for nonhematological toxicity	N/A
	164	Functional decline (binary, ADL an increase ≥2 points; IADL decrease ≥1 point), baseline vs 2-3 months	Unadjusted *P =* .352 for ADL decline Unadjusted *P =* .376 for IADL decline	N/A
Decoster^38^, 2017b	245	Mortality, median 7.9 months	Unadjusted *P =* .021	N/A
	245	Functional decline (binary, ADL an increase ≥2 points; IADL decrease ≥1 point), baseline vs 3 months	Unadjusted *P =* .849 for ADL decline Unadjusted *P =* .066 for IADL decline	N/A
Kenis^40^, 2017	387	Functional decline (binary, ADL an increase ≥2 points; IADL decrease ≥1 point), baseline vs 2-3 months	Unadjusted *P =* .075 for ADL decline Unadjusted *P =* .349 for IADL decline	N/A
Sakamoto^37^, 2021	30	Mortality, median = 5.7 months	Median overall survival = 2.5 months (not frail 0-1) vs 2.2 months (frail ≥2); *P =* .81	N/A
**Geriatric-8 (not frail >14, frail ≤14)**				
Alibhai^69^, 2021	175	Toxicity, not stated	Unadjusted *P =* .85 for chemotherapy patients (n = 104) Unadjusted *P =* .22 NAAT patients (n = 74). Note: Odds ratio reported in study but not included in meta-analysis as the reference group was not clearly reported.	N/A
Banna^58^, 2022	234	Mortality, median = 15.4 months	Adjusted HR = 3.10 (95% CI = 1.43 to 6.74)	Age, prostate-specific antigen
Beardo^59^, 2019	70	Mortality, median = 13.3 (1-48) months	Adjusted HR = 3.13 (95% CI = 1.13 to 8.66)	PS, alkaline phosphatase
Bruijnen^71^, 2022	92	Toxicity, median = 11.0 (1‐53) months	Unadjusted OR^d^ = 1.84 (95% CI = 0.62 to 5.43)	N/A
	92	Hospitalization, median = 11.0 (1‐53) months	Unadjusted OR^d^ = 2.89 (95% CI = 1.13 to 7.37)	N/A
Cavdar^70^, 2022	208	Toxicity, not stated	Adjusted OR^d^ = 2.20 (95% CI = 1.04 to 4.69)	Stage, PS, comorbidity, platinum- or taxane-based treatment regimen
	208	Treatment intolerance (cessation), 30 days after planned end of treatment	Unadjusted OR = 2.82 (95% CI = 1.04 to 7.62)	N/A
	208	Hospitalization, 30 days after planned end of treatment	Unadjusted OR = 2.83 (95% CI = 0.81 to 9.83)	N/A
Chakiba^79^, 2019	287	Functional decline (binary, ADL decrease ≥0.5), baseline vs cycle 2	Adjusted OR = 4.38 (95% CI = 1.29 to 14.92)	Age, sex
Chan^72^, 2021	259	Toxicity, up 6 months	Unadjusted OR^d^ = 0.69 (95% CI = 0.39 to 1.22)	N/A
	259	Treatment intolerance (cessation), up to 6 months	Unadjusted OR^d^ = 1.68 (95% CI = 0.81 to 3.46)	N/A
	259	Hospitalization, 6 months	Unadjusted OR^d^ = 2.03 (95% CI = 1.10 to 3.73)	N/A
Decoster^39^, 2017a	109	Toxicity, not stated	Unadjusted *P =* .102 for hematological toxicity Unadjusted *P =* .084 for nonhematological toxicity	N/A
	164	Functional decline (binary, ADL an increase ≥2 points; IADL decrease ≥1 point), baseline vs 2-3 months	Unadjusted *P =* .081 for ADL decline Unadjusted *P =* .305 for IADL decline	N/A
Decoster^38^, 2017b	245	Mortality, median = 7.9 months	Unadjusted *P =* .004	N/A
	245	Functional decline (binary, ADL an increase ≥2 points; IADL decrease ≥1 point), baseline vs 3 months	Unadjusted *P =* .205 for ADL decline Unadjusted *P =* .082 for IADL decline	N/A
Francolini^78^, 2023	93	Treatment intolerance (cessation, unrelated to progression), 6-8 cycles	Adjusted OR^e^ = 2.50 (95% CI = 1.43 to 3.33) in docetaxel subgroup Insufficient sample size to analyze cabazitaxel subgroup	Age, comorbidities
Gebbia^60^, 2021	40	Mortality, not stated	Unadjusted *P =* .0251	N/A
	40	Toxicity, not stated	Unadjusted OR^d^ hematological toxicity = 1.00 (95% CI = 0.17 to 5.67) Unadjusted OR^d^ nonhematological toxicity = 0.43 (95% CI = 0.06 to 2.90)	N/A
	40	Treatment intolerance (cessation), 2 cycles	Not statistically significant	N/A
Hamacher^61^, 2023	120	Mortality, median = 11.8 months	Adjusted HR = 1.41 (95% CI = 0.88 to 2.26)	Age, IADL, comorbidity, pazopanib, PS, liposarcoma
	118	Toxicity, median = 11.8 months	Adjusted RR = 1.54 (95% CI = 0.80 to 2.98)	Age, IADL, comorbidity, pazopanib, PS, liposarcoma
	118	Treatment intolerance (cessation, due to adverse events), median = 11.8 months	Adjusted OR = 2.34 (95% CI = ∼0.9 to 6; extrapolated from forest plot)	Age, IADL, comorbidity, pazopanib, PS, liposarcoma
Jespersen^62^, 2021	170	Mortality, not stated	Adjusted HR = 1.5 (95% CI = 1.0 to 2.4)	Age, gender, cancer type
	170	Functional decline (binary, decline in PS from 0 or 1 to ≥2), baseline vs first response evaluation	Adjusted OR = 1.5 (95% CI = 0.4 to 5.3)	Age, gender, cancer type
Kenis^40^, 2017	387	Functional decline (binary, ADL an increase by ≥2; IADL decrease by ≥1 point), baseline vs 2-3 months	Unadjusted *P =* .026 for ADL decline Unadjusted *P =* .21 for IADL decline	N/A
Kobayashi^63^, 2022	183	Mortality, median = 11 months	Unadjusted HR = 0.93 (95% CI = 0.50 to 1.72)	N/A
Kotzerke^73^, 2019	104	Toxicity, 2-3 months	Adjusted OR = 3.95 (95% CI = 1.02 to 15.38)^e^	Gender, age, body mass index, family situation, cancer stage, dose adaption during therapy, platinum-containing therapy
Li^64^, 2022	20	Mortality, median = 15 (9.25-20.25) months	Unadjusted *P =* .2097	N/A
Nakazawa^74^, 2021	93	Toxicity, median = 7.8 months	Unadjusted OR = 2.05 (95% CI = 0.66 to 6.38)	N/A
Pearce^65^, 2022	500	Mortality, median = 12.6 months (IQR = 11.9-12.9 months)	Adjusted HR = 1.85 (95% CI = 1.22 to 2.78)^e^	Age group, sex, histology, distant metastases, planned use of trastuzumab, dose reduction due to renal or hepatic function
Rier^57^, 2022	121	Mortality, median = 34 months (IQR = 16-53 months)	Adjusted HR = 1.66 (95% CI = 0.93 to 2.93)	Age, tumor type and treatment intent
	121	Treatment intolerance (cessation, not due to progression), ≥3 months	Adjusted OR = 2.34 (95% CI = 1.03 to 5.32)	Age, tumor type and treatment intent
Runzer-Colmenares^46^, 2020	496	Toxicity, not stated	Unadjusted OR^d^ = 8.18 (95% CI = 5.13 to 13.06)	N/A
Sakamoto^37^, 2021	30	Mortality, median = 5.7 months	Median overall survival = 9.3 months (not frail) vs 3.9 months (frail) *P =* .04	N/A
Von Minckwitz^76^, 2015,	317	Toxicity, not stated	adj OR = 1.82 (95% CI = 0.77 to 4.30)	Treatment arm, age, PS, body mass index, no. of meds, stage, comorbidity, IADL, VES13
	317	Treatment intolerance (cessation, premature), median = 22.8 months	Adjusted OR = 1.60 (95% CI = 0.77 to 3.35)	Treatment arm, age, PS, body mass index, no. of meds, stage, comorbidity, IADL, VES13
Wildiers^66^, 2022	79	Mortality, median = 54 months (IQR = 39.6-58.2 months)	Adjusted HR = 2.63 (95% CI = 1.11 to 6.25)^e^	Treatment, short physical performance battery
Winther^77^, 2019	160	Toxicity, median = 23.8 months (IQR = 18.8-30.9 months)	Adjusted OR = 1.08 (95% CI = 0.48 to 2.41)	Treatment arm, addition of bevacizumab, PS, no. of metastatic sites, resection of primary tumor, weight loss, carcinoembryonic antigen, c-reactive protein
	160	Treatment intolerance (cessation), 3 cycles	Unadjusted OR = 4.96 (95% CI = 1.11 to 22.10)	N/A
	160	Hospitalization, median = 23.8 months (IQR = 18.8-30.9 months)	Adjusted OR = 1.43 (95% CI = 0.64 to 3.21)	Treatment arm, addition of bevacizumab, PS, no. of metastatic sites, resection of primary tumor, weight loss, carcinoembryonic antigen, c-reactive protein
Wu^68^, 2023	127	Mortality, not stated	Median overall survival = 13.8 months (not frail) vs 7.2 months (frail) *P =* .01	N/A
	127	Toxicity, not stated	Unadjusted OR^d^ = 1.22 (95% CI = 0.52 to 2.86)	N/A
	127	Treatment intolerance (cessation), 2 cycles	Unadjusted OR^d^ = 1.03 (95% CI = 0.39 to 2.75)	N/A
	127	Hospitalization, not specified	Unadjusted OR^d^ = 1.42 (95% CI = 0.56 to 3.56)	N/A
**GA-based (fit, vulnerable, frail as per Baldicci, further groupings as specified)**				
Basso^50^, 2008	117	Mortality, median = 19 months	% alive at 2 years 37.5% for not frail (fit or vulnerable) vs 27.8% for frail; *P =* .012	N/A
	117	Toxicity, not stated	Unadjusted OR^d^ = 0.79 (95% CI = 0.36 to 1.69) for not frail (fit or vulnerable) vs frail	N/A
Brunello^51^, 2013	34	Mortality, median = 27.1 months	Unadjusted *P =* .07	N/A
	34	Toxicity, not stated	Not statistically significant	N/A
Chiusole^52^, 2023	101	Mortality, median = 32.4 months	Adjusted HR = 1.81 (95% CI = 1.02 to 3.18)^e^ for vulnerable/frail	Sex, histology, age, lung metastasis, primary tumor location, comorbidity, first-line chemotherapy, comprehensive GA score
	101	Toxicity, median = 32.4 months	Unadjusted OR^d^ = 0.84 (95% CI = 0.33 to 2.13) for vulnerable/frail	N/A
Gironés^53^, 2018	93	Mortality, not stated	Unadjusted HR = 2.52 (95% CI = 1.40 to 4.55) for vulnerable; 9.40 (95% CI = 4.82 to 18.36) for frail	N/A
	93	Toxicity, not stated	Absolute risk = 15% for fit vs 30% for vulnerable, unadjusted *P =* .0001 (risk in frail patients not reported)	N/A
Kirkhus^54^, 2017	288	Mortality, median = 16.9 (0.6-40)	Adjusted HR = 1.86 (95% CI = 1.36 to 2.56) for frail (vulnerable or frail)	Age, sex, cancer type, PS, stage, treatment
Orum^55^, 2018	189	Mortality, not stated	Unadjusted HR = 3.5 (95% CI = 1.34 to 9.15) for frail vs vulnerable (no events in fit group)	N/A
	217	Treatment intolerance (cessation, due to grade 3-4 CTCAE), 90 days	Unadjusted OR^d^ = 0.71 (95% CI = 0.16 to 3.07) for vulnerable; 0.80 (95% CI = 0.20 to 3.17) for frail	N/A
	217	Hospitalization, 90 days	Unadjusted OR^d^ = 2.56 (95% CI = 0.97 to 6.73) for vulnerable; 2.94 (95% CI = 1.16 to 7.48) for frail	N/A
Procaccio^56^, 2022	452	Mortality, not stated	Unadjusted HR = 1.41 (95% CI = 1.14 to 1.78) for vulnerable; 2.44 (95% CI = 1.91 to 3.12) for frail	N/A
Rier^57^, 2022	121	Mortality, median = 34 months (IQR = 16-53 months)	Adjusted HR = 1.37 (95% CI = 0.67 to 2.83) for vulnerable; 1.23 (95% CI = 0.58 to 2.61) for frail	Age, tumor type, treatment intent
	121	Treatment intolerance (cessation, not due to progression), ≥3 months	Adjusted OR = 0.97 (95% CI = 0.34 to 2.73) for vulnerable; 0.69 (95% CI = 0.22 to 2.2) for frail	Age, tumor type, treatment intent
**Groningen Frailty Indicator (not frail <4, frail ≥4)**				
Aaldriks^82^, 2016	493	Mortality, median = 17 (1-101) months	Adjusted HR = 1.77 (95% CI = 1.41 to 2.22)	Sex, age, purpose of treatment, type of malignancy
	494	Treatment intolerance (cessation), 4 cycles	Adjusted OR = 1.68 (95% CI = 1.08 to 2.62)	Sex, age, purpose of treatment, type of malignancy
Hamaker^86^, 2014	120	Mortality, median = 32 months	Unadjusted HR = 1.06 (95% CI = 0.61 to 1.82)	N/A
	73	Toxicity, not stated	Unadjusted OR = 1.98 (95% CI = 0.67 to 6.13)	N/A
Kroep^87^, 2015	67	Mortality, not stated	univariable *P =* .028	N/A
**VES13 (not frail <3, frail ≥3)**				
Alibhai^69^, 2021	175	Toxicity, not stated	Adjusted OR = 3.01 (95% CI = 0.81 to 11.20) for chemotherapy patients (n = 104) Unadjusted OR = 1.49 (95% CI = 0.63 to 3.57) for NAAT patients (n = 74)	Cancer aging research group toxicity risk group, gait speed (in (multivariate analysis of chemotherapy patients only)
Bauman^96^, 2012	34	Mortality, not stated	Median overall survival = 12 months in not frail vs 4.8 months in frail; unadjusted *P =* .02	N/A
Cavdar^70^, 2022	208	Toxicity, not stated	Adjusted OR = 10.06 (95% CI = 4.92 to 22.98)	Stage, PS, presence of a comorbid disease, platinum-based treatment regimen, taxane-based treatment regimen
	208	Treatment intolerance (cessation), 30 days after planned end of treatment	Unadjusted OR = 3.27 (95% CI = 1.56 to 6.85)	N/A
	208	Hospitalization, 30 days after planned end of treatment	Unadjusted OR = 4.25 (95% CI = 1.63 to 11.08)	N/A
Feliu^97^, 2021	342	Mortality, median = 24.2 (0-26.9) months	Unadjusted HR = 1.59 (95% CI = 1.01 to 2.61)	N/A
Feliu^99^, 2020	540	Toxicity, not stated	Unadjusted OR = 1.54 (95% CI = 1.02 to 2.06)	N/A
Ferrero^101^, 2018	84	Treatment intolerance (cessation), not stated	Unadjusted OR^d^ = 1.71 (95% CI = 1.13 to 2.58)	N/A
Jespersen^62^, 2021	165	Mortality, not stated	Adjusted HR = 2.1 (95% CI = 1.4 to 2.9)	Age, gender, cancer type
	165	Functional decline (binary, decline in PS from 0 or 1 to ≥2), baseline vs first response evaluation	Adjusted OR = 3.5 (95% CI = 1.0 to 11.6; note *P =* .04, hence statistically significant)	Age, gender, cancer type
Luciani^100^, 2015	648	Toxicity, not stated	Adjusted OR = 2.39 (95% CI = 1.59 to 3.60) for hematological toxicity Adjusted OR = 1.70 (95% CI = 1.06 to 2.71) for nonhematological toxicity	Hematological toxicity: sex, cumulative illness rating scale for geriatrics, PS, primary tumor site, polychemotherapy Nonhematological toxicity: PS
	648	Treatment intolerance (cessation, any cause) Follow-up: time not specified	Unadjusted OR^d^ = 1.02 (95% CI = 0.73 to 1.45)	N/A
Manokumar^102^, 2016	29	Functional decline, baseline vs 6 months	Mean Older Americans Resources and Services–IADL score difference = −0.2 for nonfrail vs -0.6 for frail (MCID 1 point); between group difference not tested	N/A
	29	HRQOL (continuous, mean FACT-G/-P score difference), baseline vs 6 months	Mean FACT-G score difference = 6.9 for nonfrail vs 5.8 for frail (MCID 4 points) Mean FACT-P score difference = 4.8 for nonfrail vs 4.5 for frail (MCID 3 points) Between group differences not tested	N/A
Ramsdale^98^, 2013	38	Mortality, not stated	Adjusted HR = 15.61 (*P =* .2)	PS, ADL, age
Runzer-Colmenares^46^, 2020	496	Toxicity, not stated	Unadjusted OR^d^ = 13.75 (95% CI = 8.26 to 22.89)	N/A
Winther^77^, 2019	160	Toxicity, median = 23.8 months (IQR = 18.8-30.9 months)	Adjusted OR = 1.45 (95% CI = 0.56 to 3.76)	Treatment arm, addition of bevacizumab, PS, no. of metastatic sites, resection of primary tumor, weight loss, carcinoembryonic antigen, c-reactive protein
	160	Treatment intolerance (cessation), 3 cycles	Unadjusted OR = 1.87 (95% CI = 0.72 to 4.85)	N/A
	160	Hospitalization, median = 23.8 months (IQR = 18.8-30.9 months)	Adjusted OR = 1.34 (95% CI = 0.55 to 3.27)	Treatment arm, addition of bevacizumab, PS, no. of metastatic sites, resection of primary tumor, weight loss, carcinoembryonic antigen, c-reactive protein

Abbreviations: ADL = activities of daily living; CFI = Carolina frailty index; CI = confidence interval; CTCAE = Common Terminology Criteria for Adverse Events; FACT (G/P) = Functional Assessment of Cancer Therapy (general or prostate); FI = frailty index; GA = geriatric assessment (based on Balducci criteria; unless specified); HR = hazard ratio; HRQOL = health-related quality of life; IADL = instrumental activities of daily living; IQR = interquartile range; MCID = minimally clinically important difference; mFI = modified Frailty Index; N/A = not applicable; NAAT = novel anti-androgen therapy; no. = number; OR = odds ratio; PS = performance status; RR = risk ratio; VES13 = Vulnerable Elders Survey-13.

a Mortality defined as overall survival; toxicity is grade 3 or higher CTCAE (unless otherwise stated); treatment intolerance is any premature cessation or modification (dose reduction) post cycle 1 (with reason, specified); functional decline and HRQOL are as defined by study; hospitalization is unplanned admission for any reason unless specified.

b Follow-up time is either planned (in days or months, or systemic anticancer treatment cycles) or actual (median [range] unless specified) from baseline.

c Hazard ratios are reported for death, and odds ratios for other adverse outcomes with 95% confidence intervals. Hazard ratios and odds ratios relate risk of negative events or outcome in frail patient groups compared with the not frail reference category.

d Calculated from raw data.

e Hazard ratios and odds ratios have been inverted to ensure that “not frail” is the reference category.

### Association between individual frailty assessment tools and outcomes

There were sufficient data available to undertake meta-analyses on the association between binary frailty assessment tools and the outcomes of overall survival, toxicity, treatment tolerance, and hospitalization, combining all frailty assessments, and then stratified by frailty tool. There were inadequate data for meta-analysis of HRQOL and/or functioning.

#### Overall survival

**Figure 2.**
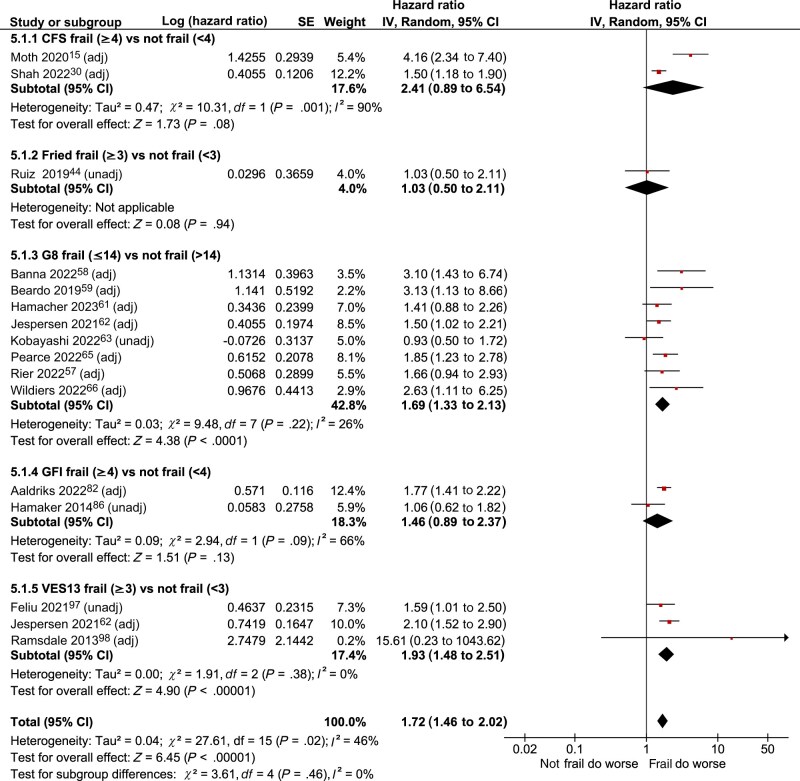
Forest plot of the association between binary frailty assessment tools and overall survival, stratified by frailty tool (primary analysis; all data). Hazard ratios relate risk of death in frail patient groups compared with the not frail reference category (scores used to categorise frail and not frail for each frailty assessment subgroup are provided in the subgroup heading). For each point estimate, the type of estimate (adjusted vs unadjusted) is specified after the study author and year. Abbreviations: adj = adjusted; CFS = clinical frailty scale; CI = confidence interval; G8 = geriatric-8; GFI = Groningen Frailty Indicator; HR = hazard ratio; unadj = unadjusted; VES13 = vulnerable elders survey-13.

Eight frailty assessment tools were evaluated in association with overall survival (Table 3), 6 of which were binary measures (CFS, Fried, fTRST, G8, GFI, and VES13). Hazard ratios for death in frail (vs not frail) patients were reported relating to all of these tools except fTRST and were synthesized in a forest plot (Figure 2). Pooled estimates for G8 and VES13 demonstrated a statistically significant association with survival (death: HR = 1.69, 95% CI = 1.33 to 2.13 and 1.93, 95% CI = 1.48 to 2.51), respectively. Pooled estimates for CFS and GFI did not demonstrate a statistically significant association with survival, though it is worth noting there were only 2 studies evaluating each of these tools. No pooling was possible for the Fried frailty score (1 study). Outside of meta-analysis, frailty assessment tools with a statistically significant association with survival in individual studies were CFS, FI-CSGA, fTRST, GA-based, and GFI (Table 3).

#### Other adverse outcomes

The number of frailty assessment tools evaluated in association with other adverse outcomes of interest were 8 toxicity, 6 treatment tolerance, 3 functional decline, 2 HRQOL, and 5 hospitalization (Table 3). Binary frailty assessment tools with evidence of statistically significant association with adverse outcomes from meta-analysis were G8 (treatment intolerance: OR = 2.25, 95% CI = 1.81 to 2.79; hospitalization: OR = 1.94, 95% CI = 1.33 to 2.81) and VES13 (grade 3 or higher toxicity: OR = 3.63, 95% CI = 1.59 to 8.32; treatment intolerance: OR = 1.67, 95% CI = 1.03 to 2.72; Figures S1-S4). Findings of all studies, including data that could be not synthesized in meta-analysis (ordinal frailty measures and/or odds ratios not available), are summarized in Table 3.

## Overall associations between frailty assessment tools and adverse outcomes

**Figure 3.**
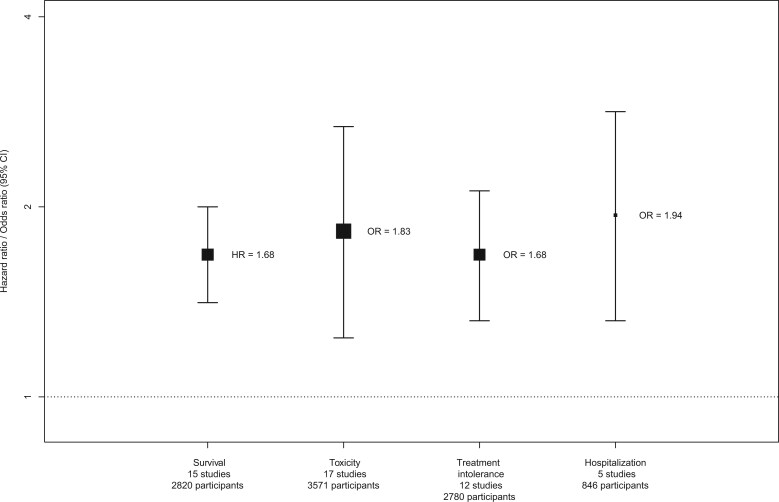
Summary of overall association between frailty (assessed using any binary tool) and adverse outcomes (secondary analysis; multiple reports accounted for). The *y*-axis outlines the 4 adverse outcomes synthesized in meta-analysis and the number of studies and participants contributing to the point estimates reported. Abbreviations: CI = confidence interval; HR = hazard ratio; OR = odds ratio.

A secondary analysis was undertaken to provide overall pooled estimates for the association between the presence of frailty (assessed using any binary tool) and outcomes, after accounting for multiple reports. In this meta-analysis, overall pooled estimates demonstrated that frail (vs not frail) patients had a statistically significant increased risk of all adverse outcomes evaluated (death: HR = 1.68, 95% CI = 1.41 to 2.00; toxicity, treatment intolerance, and hospitalization: OR = 1.83, 95% CI = 1.24 to 2.68, OR = 1.68, 95% CI = 1.32 to 2.12; and OR = 1.94, 95% CI = 1.32 to 2.83, respectively; Figure 3 and Figures S5-S8).

### Sensitivity analyses and heterogeneity

Sensitivity analyses exploring the impact of various methodological and clinical factors on overall pooled estimates and statistical heterogeneity are presented in Table S2.

Sensitivity analyses demonstrated that removing studies with high risk of bias, those that did not adjust for covariates, and those not focusing on the advanced disease and/or palliative settings had no impact on the main findings (the positive statistically significant association between frailty and outcome remained in all of these analyses). In the sensitivity analysis where only adjusted data were included, the hazard ratio (number of studies contributing) for survival was 1.95 (95% CI = 1.62 to 2.35; 10 studies), and odds ratios for toxicity and treatment intolerance were 1.99 (95% CI = 1.43 to 2.78; 7 studies) and 2.18 (95% CI = 1.76 to 2.70; 5 studies). There were too few studies for a sensitivity analysis looking at adjusted data only for the hospitalization outcome. Similarly, there were too few studies in specific tumor sites to explore the impact of tumor site on pooled estimates in detail, but this was attempted in the most common tumor site (GI). In GI studies, statistical significance remained for survival (where 5 studies contributed to the analysis) but not for toxicity (3 studies). There were an inadequate number of GI studies for a sensitivity analysis of treatment tolerance and hospitalization outcomes.

As expected across clinically heterogenous populations, statistical heterogeneity exists between studies. Within studies contributing to overall pooled estimates, statistical heterogeneity (*I*^2^) was high for the outcome of toxicity (88%), moderate for overall survival (46%) and treatment tolerance (48%), and low for hospitalization (0%). Sensitivity analyses demonstrate that some statistical heterogeneity was related to methodological heterogeneity (*I*^2^ for toxicity fell from 88% to 17% when studies only reporting unadjusted estimates were excluded), and some were related to clinical heterogeneity (*I*^2^ for overall survival fell from 46% to 12% in sensitivity analyses where only studies of patients with GI cancers were included, and *I*^2^ for treatment tolerance fell from 48% to 0% in sensitivity analysis where only studies focusing on the advanced disease and/or palliative setting were included).

Funnel plots exploring publication bias and small study effects showed no evidence of asymmetry (Figures S9-S12).

## Discussion

Patients with cancer undergoing systemic anticancer treatment who are identified as frail are at considerably increased risk of mortality (HR = 1.68, 95% CI = 1.41 to 2.00), toxicity (OR = 1.83, 95% CI = 1.24 to 2.68), treatment intolerance (OR = 1.68, 95% CI = 1.32 to 2.12), and hospitalization (OR = 1.94, 95% CI = 1.32 to 2.83) compared with those who are deemed not frail. These statistically significant associations persisted when only studies that adjusted for confounding variables were included in the analyses, supporting the notion that these key meta-analytic findings are not unduly influenced by confounding factors. Two individual frailty assessment tools had a statistically significant association with outcomes in the meta-analyses: G8 with survival, treatment tolerance, and hospitalization and VES13 with survival, toxicity, and treatment tolerance. To our knowledge this is the first meta-analysis of the association between frailty assessment tools and treatment outcome in adults undergoing systemic anticancer treatment. Our findings provide robust evidence that frailty is prognostic for a range of systemic anticancer treatment outcomes that are important to patients and clinicians.

Our study adds weight to the evidence around the role of frailty assessments in patients undergoing systemic anticancer treatment, which to date has come primarily from individual studies and narrative syntheses. A recent (2023) narrative review by Goede^12^ identified 14 systematic reviews examining frailty screening and geriatric assessment in older adults undergoing a range of cancer treatments. These reviews provide evidence of the sensitivity and specificity of frailty screening for identifying cancer patients likely to benefit from geriatric assessment^11^^,^^103^^,^^104^ and the prognostic value of frailty as assessed by geriatric assessment.^2^ However, high-quality pooled synthesis of studies exploring the prognostic value of frailty assessment tools (via either geriatric assessment–based or briefer screening tools) in patients undergoing specific treatments has been lacking. A review by Garcia et al.^104^ in 2021 found G8 and VES13 to be the most frequently evaluated frailty screening tools in cancer populations, with G8 having higher sensitivity and VES13 having higher specificity. The only review summarizing evidence on the prognostic value of a frailty screening tool was published by van Walree et al.^11^ and explored the G8 screening tool in patients undergoing a range of therapies, including chemotherapy. They found G8 to be prognostic for survival and treatment-related complications but that it was less frequently studied in association with treatment completion, health-care utilization, and patient-centered outcomes.

We have systematically synthesized all evidence on the prognostic value of any validated frailty assessment tool in adults undergoing systemic anticancer treatment. We identified 58 articles exploring 9 tools, including a number of validated frailty screening tools, which may have advantages over traditional geriatric assessment–based tools in terms of feasibility of implementation in routine practice. We were able to generate the first pooled estimates for the association between frailty and survival, toxicity, treatment tolerance, and hospitalization. We build on the 2019 findings of van Walree et al.^11^ and provide robust evidence from pooled analysis that G8 and VES13 are prognostic for survival and treatment-related complications. Our review compiles evidence on the association between several other less well studied frailty tools and outcomes of interest for the first time. It is important to note that while G8 and VES13 are more widely studied, this does not necessarily mean they are any better for assessing frailty in practice than other less well studied tools.

The narrative review by Goede^12^ highlights an increasing interest in CFS, which is arguably one of the simplest tools for assessing frailty, and identifies 7 studies demonstrating its prognostic value in cancer populations. The majority were undertaken in surgical settings, with only 1 (Pearce et al.^65^) focusing on adults undergoing systemic anticancer treatment. Although data from Pearce et al.^65^ regarding G8 and outcome are included in our synthesis, data on CFS were not eligible as the authors employed a modified CFS derived from patient-reported outcome data. Frailty assessments focusing largely on patient functioning, including CFS (usually clinician assessed) and VES13 (patient completed and based on activities of daily living (ADL) and instrumental ADL items) appear to perform well in this review; this is in keeping with the findings of a previous review demonstrating that functional status (via ADL and/or instrumental ADL) is prognostic in older adults with cancer.^105^

There are several strengths and limitations in relation to the design of this review that should be considered when interpreting its findings. The absence of limitations to searches in terms of year or language of publication helps minimize inclusion bias. Although no filters for age were used, most included studies did focus specifically on older patients; this can be considered a considerable strength of this work, as older adults are often excluded from research, and studies specifically focusing on their care are lacking.^106^^,^^107^ This also valuably demonstrates a lack of research into the prognostic value of frailty assessments in younger adults undergoing systemic anticancer treatment. The use of frailty assessments in younger patients is an important area for future research, particularly as frailty and chronic conditions including cancer are more prevalent and can coincide in younger patients living in areas of greater deprivation.^108^ Beyond patients’ age and tumor characteristics and study country, it was difficult to evaluate the demographic mix included within this review as ethnicity and sociodemographic factors were seldom reported in included studies.

It is recognized that there is statistical heterogeneity across the studies synthesized in meta-analysis, but sensitivity analyses demonstrate that this is explained by a combination of clinical and methodological factors. Reassuringly, the positive association between increasing frailty and risk of adverse outcomes is consistent across the analyses. The inclusion of studies evaluating patients with any nonhematological malignancy undergoing systemic anticancer treatment demonstrates the broad applicability of frailty assessments across different tumor sites in patients undergoing this specific treatment, particularly in the advanced disease and/or palliative treatment-intent setting. Although studies of patients with any treatment intent were considered eligible, many studies in the adjuvant/neoadjuvant setting were excluded because of the timing of frailty assessments (not clearly at baseline prior to systemic anticancer treatment). Many eligible studies included a mixture of disease stages and/or treatment intents, but only 2 focused on patients being treated with curative intent, which may limit the applicability of review findings to such populations.

In this review and meta-analysis, studies were only deemed eligible for inclusion if they reported on a validated frailty tool and provided a definition for frailty; the practical application of this meant that frailty must have been categorized rather than reported only as a continuous score. For consistency, we considered frailty assessment tools that had been validated in any setting as potentially eligible^109^ and the cut-points for frailty as defined in initial validation studies (where applicable). We excluded studies that made important changes to tools or cut-points for defining frailty. This improves the standardization of included tools but may mean that some frailty tools (particularly various frailty indices and geriatric assessment–based tools adapted to different settings) and cut-points, which may well have prognostic value, are not fully represented in this review. For example, since its initial development,^8^ work exploring the G8 tool in GI cancer patients has suggested that it may overestimate frailty in this setting because of high weighting of nutrition questions. Lower cut-points have been suggested,^77^^,^^110^^,^^111^ but for consistency, data from studies evaluating different cut-points are not included in this review. Synthesis of continuous frailty score data was beyond the scope of this review, but scores inevitably provide more granular information about a patients’ frailty than an ordinal or binary category. Estimates for the association between frailty categories and outcome provide a starting point for informing decision making, but continuous scores should also be considered. As clinicians and teams become more familiar with a specific tool in their setting, they will develop a deeper understanding of how frailty scores relate to decision making and outcomes for their patients.

Although this review provides good evidence about the association between frailty and traditional outcomes of survival, toxicity, and hospitalization, consistent with the findings of van Walree,^11^ we found less research looking at patient-centered outcomes of functional decline and quality of life. Furthermore, there are other outcomes that are important to patients undergoing systemic anticancer treatment that were beyond the scope of this review. A key example is cognitive outcomes, which are known to be affected by systemic anticancer treatment and have a considerable impact on QOL. The association between frailty and cognitive outcomes is an important area for future research.

Regarding quality of included studies and bias, reassuringly most were at low or moderate risk of bias, and sensitivity analyses demonstrated that excluding studies with high risk of bias did not change the overall pooled estimates. Systematic reviews of observational studies are prone to publication bias,^10^ but funnel plots demonstrated no concerning asymmetry in our synthesis. A key limitation of much of the published literature contributing to our review is the lack of adjustment for confounding. Our decision to summarize adjusted and unadjusted risk estimates allowed us to make best use of all the available evidence while ensuring that each point estimate is clearly labeled to specify whether this was adjusted or not. By its nature, frailty is multifactorial and associated with a range of confounding variables including patient age and comorbidities. Lack of adjustment for such factors may mean studies overstate the association between frailty and outcome. To address this, sensitivity analyses were undertaken, which reassuringly demonstrated that even when limiting the analysis to only the small number of studies that adjusted for confounding variables, the main findings in terms of the direction and statistical significance of the association between frailty and outcome remained (Figures S5-S8)

The literature on frailty in cancer settings and the association between frailty and outcome has grown considerably over recent years, and indeed further studies have been published in the period since the searches were run, and future reviews will be valuable to address some of the limitations of the literature identified in this review.

Our findings will help clinicians understand the prognostic value of frailty assessment findings within systemic anticancer treatment decision making and may be used to inform the selection of frailty assessment tools for use within clinical trials and practice. Clinicians and teams considering utilizing frailty assessments within their practice can consult our findings to appraise the existing evidence on the prognostic value of existing tools and its external validity to their setting (disease site and treatment intent) and should weigh this alongside the feasibility of implementation in their practice. Robust estimates of the increased risk of important adverse outcomes from this review should be used alongside other clinical factors and patient preferences to directly support treatment decision making. Questions remain about the practicalities of using frailty assessments within decision making and particularly shared decision making with patients, and practical tools are required. To address this, in the next steps of this research, a frailty-informed cancer management intervention is being codesigned with key stakeholders (patients and caregivers, health-care professionals, and academic experts) to support the use of frailty assessments within shared decision making for older patients with advanced cancer.^112^

We provide robust evidence that patients with frailty undergoing systemic therapy are at considerably increased risk of adverse outcomes during systemic anticancer treatment, including death, toxicity, treatment intolerance, and unplanned hospitalization. Frailty assessments and associated risk estimates should be considered alongside other clinical factors and patient preferences to support shared decision making, particularly for older adults with advanced cancer and/or being treated with palliative intent. Further work is needed to understand the role of frailty assessments in younger adults and those undergoing curative-intent treatment.

Evidence from this review will be valuable to inform the selection of frailty assessments for use within clinical trials and practice. Teams deciding which tool to use in their setting should weigh the benefits of the tool in terms of the evidence of prognostic value in their context alongside the feasibility and resources required for implementation.

## Supplementary Material

djaf017_Supplementary_Data

## Data Availability

The data contributing to this review can be accessed via included study reports, which are referenced within the article. Full details of the new data generated from meta-analysis are available in the article and within its online supplementary material. Any further information is available on request by contacting the corresponding author.
