# Supplementary material for: Frailty and outcomes in adults undergoing systemic anticancer treatment: a systematic review and meta-analysis
Source: J Natl Cancer Inst. 2025 Jan 30;117(7):1316–39. doi: 10.1093/jnci/djaf017 (PMC12232047; doi:10.1093/jnci/djaf017)
Supplement: djaf017_Supplementary_Data [file djaf017_supplementary_data.pdf]

Supplementary Materials

Contents

Supplementary Methods 3

Supplementary Methods 1. Study characteristics/selection criteria and definitions 3

Supplementary Methods 2. Example of search strategy 5

Supplementary Methods 3. Data extracted from included studies 7

Supplementary Methods 4. Sources and handling of multiple reports 9

Supplementary Methods 5. Decision rules 11

Supplementary Methods 5.1. Summary statistic decision rules 11

Supplementary Methods 5.2. Outcome-specific decision rules 11

Supplementary Tables 13

Supplementary Table 1. Risk of Bias (QUIPS) assessments 13

Supplementary Table 2. Summary of additional analyses, including sensitivity analyses 16

Supplementary Figures: Forest plots for primary meta-analysis (all data) 18

Supplementary Figure 1. Forest plot of the association between binary frailty assessment tools and overall survival (primary analysis, all data, stratified by frailty tool) 19

Supplementary Figure 2. Forest plot of the association between binary frailty assessment tools and grade 3+ toxicity (primary analysis, all data, stratified by frailty tool) 20

Supplementary Figure 3. Forest plot of the association between binary frailty assessment tools and treatment intolerance (primary analysis, all data, stratified by frailty tool) 21

Supplementary Figure 4. Forest plot of the association between binary frailty assessment tools and hospitalisation ((primary analysis, all data, stratified by frailty tool) 22

Supplementary Figures: Forest plots for secondary meta-analysis (multiple reports accounted for) 23

Supplementary Figure 5. Forest plot of the association between binary frailty assessment tools and overall survival (secondary analysis, multiple reports accounted for) 23

Supplementary Figure 6. Forest plot of the association between binary frailty assessment tools and grade 3+ toxicity (secondary analysis, multiple reports accounted for) 24

Supplementary Figure 7. Forest plot of the association between binary frailty assessment tools and treatment intolerance (secondary analysis, multiple reports accounted for) 25

Supplementary Figure 8. Forest plot of the association between binary frailty assessment tools and hospitalisation (secondary analysis, multiple reports accounted for) 26

Supplementary Figures: Funnel plots for primary meta-analysis (all data) 27

Supplementary Figure 9(a). Funnel plot survival (primary analysis, all data) 27

Supplementary Figure 9(b). Funnel plot – survival (primary analysis, Ramsdale 2013 outlier removed) 28

Supplementary Figure 10. Funnel plot toxicity (primary analysis, all data) 29

Supplementary Figure 11. Funnel plot treatment intolerance (primary analysis, all data) 30
Supplementary Figure 12. Funnel plot hospitalisation (primary analysis, all data).........................31

Supplementary Methods

Supplementary Methods 1. Study characteristics/selection criteria and definitions

Full inclusion/exclusion criteria are outlined in the table below, which further defines the required study Population, Intervention, Outcome, Setting and Timing of prognostic factor/outcome assessment (based on the PICOTS typology (1))

PICOTS item	Inclusion*(/exclusion)* criteria for PICOTS item
POPULATION	Adults (aged ≥18) with solid-organ malignancy (any non-haematological primary; any stage or treatment intent) undergoing (or being considered for) SACT (including cytotoxic chemotherapy, immunotherapy and targeted treatments)<br><br>*Exclusion criteria:*<br>• *Studies where SACT is not the predominant treatment being evaluated (i.e. patients are receiving hormones alone, or combination therapy where the main treatment being evaluated is radiotherapy or surgery)*<br>• *Studies where patients being considered for SACT are included (potentially alongside patients being considered for other treatments), but <50% of participants go on to receive SACT*<br>• *Studies where patients are systematically treated differently based on their level of frailty*
INTERVENTION (PROGNOSTIC FACTOR)	Any validated frailty assessment measure (defined as: any validated assessment tool that can be used to provide an overall assessment of patient frailty within clinical practice)<br><br>*Exclusion criteria:*<br>• *Studies evaluating tools that have not undergone previous validity testing*<br>• *Studies evaluating biochemical, physical or radiological markers of frailty/sarcopenia*<br>• *Studies evaluating tools that do not provide a definition for frailty based on the evaluated tool (e.g. studies including assessment of geriatric assessment domains that do not categorise patients according to their overall level of frailty, and toxicity/mortality prediction tools for which the output is a risk category)*<br>• *Studies which are evaluating a validated frailty assessment tools but have made changes to the tool and/or definition/cutpoint for frailty provided in validation studies*
OUTCOME	Any of the following measures of systemic anti-cancer treatment outcome:<br>• Overall survival (from baseline diagnosis, frailty assessment or first cycle of SACT to death from any cause; main outcome of interest)<br>• Toxicity (CTCAE-graded; grade 3+ and/or any grade)

	• Treatment tolerance (proxy measures: treatment discontinuation, modification or delay after 1st cycle)<br>• Health-related quality of life (assessed using a validated generic or disease-specific tool)<br>• Functional decline (any measure, including decline in patient or clinician-reported functioning, or new loss of independent functioning/requirement for care e.g. home care or move to residential/nursing home)<br>• Unplanned hospitalisation (unplanned/unexpected/non-elective attendance/admission for any reason)<br><br>*Exclusion criteria:*<br><br>*1. Studies where no outcomes meeting criteria above are reported*
TIMING	Prognostic factor: Measured at baseline (before commencing SACT)<br><br>Outcome: Any follow-up period after first cycle of SACT (will inevitably vary by outcome measure)<br><br>*Exclusion criteria:*<br><br>*1. Studies where frailty assessments are not undertaken at baseline (prior first SACT cycle), or where they are undertaken earlier in the patient pathway e.g. prior to surgery in the adjuvant setting*<br>2. *Studies without eligible outcomes assessed the after first cycle of SACT*
SETTING	Any outpatient setting (primary or secondary care; community or hospital)<br><br>*Exclusion criteria:*<br><br>1. *Studies where recruitment occurred and/or frailty was assessed during an acute admission*

References

1. Matchar DB. Introduction to the Methods Guide for Medical Test Reviews. In: Chang SM, Matchar DB, Smetana GW, et al., editors. Methods Guide for Medical Test Reviews [Internet]. Rockville (MD): Agency for Healthcare Research and Quality (US); 2012 Jun. Chapter 1. Available from: https://www.ncbi.nlm.nih.gov/books/NBK98245/ [Accessed 25/03/24]

Supplementary Methods 2. Example of search strategy

Example of search strategy (key concepts and search terms, as ran in OVID Embase)

**Key concepts**	**Search terms**
Cancer	1. exp neoplasms/<br>2. (neoplasm* or cancer* or carcinoma* or tumo?r* or malignan* or metasta*).tw.<br>3. or/1-2 [cancer]
Frailty	4. (frail* adj5 (instrument * or tool* or index* or score* or scale* or measur* or assess* or test* or screen* or marker*)).tw.<br>5. (Rockwood adj3 (scale* or scor* or CFS)).tw.<br>6. Groningen Frailty Indicator.tw.<br>7. (Fried adj3 (criteria or phenotype*)).tw.<br>8. (Geriatric 8 or Geriatric8).tw.<br>9. ((G8 or G 8) adj3 (scor* or screen*)).tw.<br>10. Vulnerable Elder* Survey.tw.<br>11. (VES 13 or VES13).tw.<br>12. geriatric assessment/<br>13. geriatric assessment*.tw.<br>14. or/4-13 [frailty]
Prognostic factors	15. Cohort analysis/<br>16. incidence.tw.<br>17. mortality/<br>18. follow up/<br>19. prognos*.tw.<br>20. prognosis/<br>21. Predict*.tw.<br>22. course.tw.<br>23. survival analysis/<br>24. risk factor/<br>25. or/15-24 [prognosis factor]
SACT	26. (chemotherap* or SACT or systemic anticancer treatment* or immunotherap* or targeted treatment* or targeted therap* or tyrosine kinase inhibitor* or antineopastic* or anti-neoplastic*).tw.

	27. ((anticancer* or anti-cancer* or antitumo?r* or anti-tumo?r* or anticarcinogen* or anticarcinogen*) adj4 (drug* or agent* or therap* or treat* or medicat* or protocol*)).tw.<br>28. Drug Therapy/<br>29. exp "Antineoplastic Agents"/<br>30. exp cancer chemotherapy/<br>31. or/26-30 [SACT]
All	32. 3 and 14 and 25 and 31

Supplementary Methods 3. Data extracted from included studies

Data extracted from included studies is summarised in the table below. Selection of data items to extract were informed by the Checklist for critical Appraisal and data extraction for Systematic Reviews of Prediction Modelling Studies (CHARMS) checklist[1]. Where full texts/supplementary information was unavailable or data missing, inconsistent or unclear, authors were contacted to request the full manuscript/supplementary information, missing information or clarification.

Domain	Key items
Study ID	First author<br>Year of publication
Source of data	Study design: Case-control vs cohort study<br>Is data from a clinical trial?
Participants	Eligibility and recruitment method (location, number of centres, setting, inclusion and exclusion criteria)<br>Participant description:<br>• Age (mean with SD and/or median with IQR)<br>• Sex (male/female; %)<br>• Performance status (% with each score and/or PS category)<br>• Frailty (% in each frailty category)<br>• Tumour site (% in each category)<br>• Disease staging/intent (% in each category)
Intervention/comparison index test	Index frailty tool(s) assessed<br>For each frailty tool: definition, method and timing of assessment (including how 'fit' vs 'frail' patients are defined within the study/ analyses)
Outcome(s) assessed	Outcome(s) used in study including:<br>• Overall survival<br>• Treatment tolerance and/or toxicity<br>• Unplanned hospitalisation<br>• Functional decline<br>• Quality of life<br><br>For each outcome: Definition, method and timing of measurement. Was the same outcome definition and method of measurement used in all patients? Was the outcome assessed without knowledge of the candidate predictors?
Sample size	Number of participants and number of outcomes/events
Missing data	Number of participants with any missing value (including index test/outcome data)

	Handing of missing data (e.g., complete-case analysis, imputation, or other methods)
Results	Binary outcomes (yes/no; number of events/total number in population) for ‘fit’ (‘best’ scores) vs ‘frail’ (‘poorest’ scores) patients:<br>• Mortality (all cause)<br>• Toxicity (any, grade 1-2 and/or grade 3-4)<br>• Treatment stopped after #1 due to intolerance<br>• Treatment plan modified (e.g. delay, dose adjustment) after #1 due to intolerance<br>• Unplanned hospital admission<br>• Loss of independent function (e.g. care requirement, care home admission)<br>Continuous outcomes for ‘fit’ vs ‘frail’ patients (mean, SD/SE and 95% CI):<br>• Overall survival (length of survival, weeks)<br>• Health status (quality of life; change from or adjusted for baseline)<br>• Duration hospitalised due to treatment-related effects (days)<br>• Functional status (ADL/IADL/PS; change from or adjusted for baseline)<br><br>Between group comparisons: unadjusted and adjusted outcome data for ‘fit’ vs ‘frail’ patients (with 95% confidence intervals (CIs) and p-values):<br>• Risk ratios (RR) and odds ratios (OR) dichotomous outcomes<br>• Hazard ratios (HR) for time-to-event data<br>• Difference in outcome for continuous measures<br>For all outcomes: follow-up length and covariates adjusted for.

References

1. Moons KGM, de Groot JAH, Bouwmeester W, et al. Critical Appraisal and Data Extraction for Systematic Reviews of Prediction Modelling Studies: The CHARMS Checklist. PLOS Medicine. 2014;11(10): e1001744.

Supplementary Methods 4. Sources and handling of multiple reports

Many included studies in this review report on a mixture of frailty assessments and outcomes, sometimes in more than one article. Potential sources of multiple reports have been identified in this review and careful consideration has been taken to how these are handled to minimise multiple reporting biasing our review findings. Sources and handling of multiple reports are summarised in the table below.

Potential source of multiple reports	Handling of multiple reports in tabulated results	Handling of multiple reports in meta-analysis (by frailty tool and overall)
Multiple reports from a single study	Only a single article/report from each study (the one with the largest sample size and/or longest follow-up period) contributed to the tabulated results for each outcome	Only a single article/report from each study (the one with the largest sample size and/or longest follow-up period) contributed to the meta-analysis for each outcome
Multiple frailty assessment tools reported on in a single study/report	It is accepted that some studies may report on the prognostic value of more than one frailty tool. Data relating to all eligible validated frailty assessment tools were extracted and reported in the tabulated results for each outcome.	Data on all frailty assessment tools reported upon in each study are included in the primary meta-analysis to give accurate pooled estimates for association between each frailty tool and outcome (frailty assessment subgroup pooled estimate).<br>To avoid multiple reports biasing the overall pooled estimates of the association between frailty and each outcome, a secondary analysis was undertaken where the point estimate for only one frailty tool contributed (as described in **Supplementary Table 2**). Findings from this secondary analysis provide the main results for the overall association between frailty and each outcome (**Supplementary Table 2 and Supplementary Figures 5-8**.)
Multiple outcome measures are reported on in a single study/report	It is accepted that some studies may report on more than one outcome of interest. Data relating to all outcomes of interest were extracted and reported in the tabulated results. However, where studies	Only a single outcome measure contributes to the meta-analyses for each outcome from each study.

	report on more than one specific measure for an outcome of interest (e.g. more than one proxy for treatment intolerance), decision rules are applied to ensure that only a single outcome measure contributes per outcome per study (as described in **Supplementary Methods 5**).	

Methods for handling multiple reports described above seek to minimise their impact on the key findings of this review in terms of the estimates of association between frailty and outcome. However, it must be recognised that there are some studies that report on multiple frailty assessment tools/outcomes that inevitably contribute more data to this review overall; these can be identified visually by consulting the included studies table (**Table 1**).

Supplementary Methods 5. Decision rules

Supplementary Methods 5.1. Summary statistic decision rules

Decision rules were applied to ensure that summary statistics reported in the tabulated summary of results within this review were selected systematically and minimise reporting bias. The core decision rules which determined the summary statistic/metric reported for each outcome are as follows:

- 1st choice: preferred adjusted ratio (HR for survival, OR for other outcomes)
- 2nd choice: preferred unadjusted ratio (calculated from raw data if required)
- 3rd choice: other ratio (adjusted if available, otherwise unadjusted)
- 4th choice: continuous measure (adjusted if available, otherwise unadjusted)
- 5th choice: p-value if available, or narrative statement of statistical significance

Supplementary Methods 5.2. Outcome-specific decision rules

Additional outcome-specific decision rules were used for selecting which outcome measure to report where studies may report more than one (this applies to all outcomes other than overall survival). Decision rules were determined a priori (before commencing meta-analysis) and took into consideration available data as well as which outcomes were likely to represent the most substantial impact on a patient's life overall. The outcome-specific decision rules for each outcome are described below.

Toxicity outcome selection:

- 1st choice: CT CAE grade 3+ toxicity (all types; if haem/non-haem are only reported separately, both are included in the tabulated results and the largest point estimate is included in the meta-analysis)
- 2nd choice: CT CAE any grade toxicity (all types)

Note: all studies except one reported on the 1st choice outcome measure for toxicity (CT CAE grade 3+). Studies that only reported on a single toxicity type (e.g. neutropenia, fatigue) were excluded from this review.

Treatment intolerance outcome selection:

- 1st choice: treatment stopped (permanent cessation) post #1
- 2nd choice: treatment modification (e.g. dose reduction) post #1
- 3rd choice: treatment delay/interruption post #1

Note: all studies reported on the 1st and/or 2nd choice outcome measure for treatment intolerance hence there is no data in the tabulated results relating to treatment delays.

Functional decline outcome selection:

- 1st choice: new loss of independent functioning/requirement for care
- 2nd choice: decline in validated patient reported measure of functioning e.g. ADL/IADL (both will be reported)
- 3rd choice: decline in validated clinician assessed measure of functioning (ECOG performance status)

Note: no studies reported on the 1st choice outcome measure; all studies reported on patient or clinician reported functioning (2nd/3rd choice).

Health-related Quality of Life outcome selection:

- 1st choice: any validated generic or disease specific health-related quality of life measure (all are reported in the table)

Note: there were only 2 studies reporting on health-related quality of life beyond functioning. Given the scarcity of evidence, all available data is reported.

Hospitalisation outcome selection:

- 1st choice: unplanned/acute hospital admission for any reason
- 2nd choice: unplanned/acute hospital admission due to toxicity / not due to progression
- 3rd choice: other unplanned attendance (e.g. visit to emergency department)

Note: most reported on the 1st and/or 2nd choice outcome measure for hospitalisation.

Supplementary Tables

Supplementary Table 1. Risk of Bias (QUIPS) assessments

Authors	Year	1. Study Participation	2. Study Attrition	3. Prognostic Factor Measurement	4. Outcome Measurement	5. Study Confounding	6. Statistical Analysis and Reporting	**OVERALL RISK OF BIAS**
Aaldriks	2011	high	high	mod	low	low	low	**mod**
Aaldriks	2013a	mod	mod	low	high	mod	mod	**mod**
Aaldriks	2013b	low	high	mod	mod	low	mod	**mod**
Aaldriks	2016	mod	mod	mod	mod	low	low	**mod**
Akbıyık	2022	mod	mod	low	high	mod	mod	**mod**
Alibhai	2021	low	low	high	mod	mod	low	**mod**
Banna	2022	mod	mod	mod	high	low	mod	**mod**
Basso	2008	low	mod	low	mod	mod	low	**mod**
Bauman	2012	low	high	high	high	mod	mod	**high**
Beardo	2019	mod	mod	high	mod	mod	low	**mod**
Bruijnen	2022	mod	mod	mod	low	mod	high	**mod**
Brunello	2013	high	mod	high	mod	high	high	**high**
Cavdar	2022	low	low	low	low	low	mod	**low**
Chakiba	2019	mod	low	low	mod	low	mod	**low**
Chan	2021	low	low	mod	low	mod	mod	**low**
Chiusole	2023	low	high	mod	low	low	low	**mod**
Decoster	2017a	low	low	low	mod	mod	mod	**mod**
Decoster	2017b	low	high	low	low	mod	low	**low**
Feliu	2021	low	mod	mod	mod	mod	low	**mod**
Feliu	2020	mod	mod	low	low	mod	mod	**mod**

Ferrero	2018	mod	mod	low	mod	mod	mod	**mod**
Francolini	2023	high	high	low	low	mod	mod	**mod**
Gebbia	2021	low	mod	high	mod	mod	mod	**mod**
Gironés	2018	mod	high	mod	high	mod	mod	**mod**
Hamacher	2023	low	mod	high	high	mod	low	**mod**
Hamaker	2014	low	mod	mod	low	mod	mod	**mod**
Hay	2019	low	mod	mod	low	mod	mod	**mod**
Jespersen	2021	mod	mod	mod	mod	low	low	**mod**
Kenis	2017	low	low	mod	low	mod	low	**low**
Kirkhus	2017	mod	mod	mod	low	low	low	**mod**
Kobayashi	2022	low	high	mod	high	mod	low	**mod**
Kotzerke	2019	low	mod	mod	mod	mod	low	**mod**
Kroep	2015	low	low	mod	mod	low	low	**low**
Li	2022	low	high	mod	low	mod	low	**low**
Luciani	2015	high	high	mod	high	high	mod	**mod**
Manokumar	2016	low	mod	low	high	mod	high	**mod**
Mathur	2022	low	mod	high	mod	mod	mod	**mod**
Moth	2020	mod	high	mod	mod	mod	mod	**mod**
Nakazawa	2021	mod	high	mod	mod	mod	low	**mod**
Orum	2018	mod	high	high	mod	mod	high	**high**
Pearce	2022	mod	mod	low	mod	low	low	**low**
Phaibulvatanapong	2018	mod	mod	mod	mod	mod	mod	**mod**
Procaccio	2022	high	high	high	mod	mod	low	**high**
Ramsdale	2013	high	high	high	high	mod	high	**high**
Rier	2022	low	high	mod	mod	low	mod	**mod**
Rittberg	2021	mod	high	high	low	high	low	**high**
Ruiz	2019	low	mod	high	low	high	mod	**mod**
Runzer-Colmenares	2019	UTA	UTA	UTA	UTA	UTA	UTA	**UTA**
Runzer-Colmenares	2020	mod	low	low	mod	high	mod	**mod**
Sakamoto	2021	low	low	mod	low	mod	low	**low**

Shachar	2022	low	mod	low	mod	mod	mod	**mod**
Shah	2022	mod	mod	low	mod	low	mod	**mod**
Von Minckwitz	2015	low	mod	low	mod	mod	low	**mod**
Weiss	2020	mod	mod	high	low	mod	mod	**mod**
Wildiers	2022	low	high	high	high	mod	mod	**high**
Wildiers	2018	low	low	mod	low	mod	low	**low**
Winther	2019	low	low	mod	low	mod	low	**low**
Wu	2023	mod	mod	mod	mod	mod	low	**mod**
Total low risk of bias		29	11	16	19	13	25	**11**
Total moderate risk of bias		22	28	27	27	39	27	**39**
Total high risk of bias		6	18	14	11	5	5	**7**
Total unable to assess (UTA)*		1	1	1	1	1	1	**1**
Total number of studies		58	58	58	58	58	58	**58**

Footnotes:
*Runzer-Colmenares (2019) is a Spanish language study; it was possible to extract basic demographic details about the study from the abstract to facilitate its inclusion in this review but full translation to facilitate RoB assessment was not possible.
Abbreviations/acronyms: mod = moderate

Supplementary Table 2. Summary of additional analyses, including sensitivity analyses

In addition to the primary ('frailty assessment subgroup pooled estimate') meta-analysis, number of additional analyses were undertaken, including:

- A secondary ('overall pooled estimate') meta-analysis which accounts for multiple reports and provides more robust estimates for the overall association between frailty and outcome
- A number of sensitivity analyses undertaken to explore and demonstrate the impact of study factors (duplicity, risk of bias and adjustment for covariates) and clinical factors (GI tumour site, disease stage/treatment intent) on the overall pooled estimates for each outcome and statistical heterogeneity (I^2).

The findings of the additional analyses undertaken are summarised in the table below. **Supplementary Figures 5-8** are the forest plots from which the secondary meta-analysis data is derived**.**

	**Treatment outcome**<br>No. of studies reporting on outcome<br>No. of studies contributing to reports on multiple frailty assessments within primary meta-analysis and details (author/year: frailty assessment tools contributed)			
Analysis	**Survival**<br>Studies: 15<br>Studies contributing multiple frailty assessments: 1 (Jespersen 2021: G8/VES13)	**Toxicity**<br>Studies: 17<br>Studies contributing multiple frailty assessments: 3 (Runzer-Colmenares 2020: Fried/G8/VES13; Cavdar 2022: G8/VES13; Winther 2019: G8/VES13)	**Treatment intolerance**<br>Studies: 12<br>Studies contributing multiple frailty assessments: 2 (Cavdar 2022: G8/VES13; Winther 2019: G8/VES13)	**Hospitalisation**<br>Studies: 5<br>Studies contributing multiple frailty assessments: 2 (Cavdar 2022: G8/VES13; Winther 2019: G8/VES13)
**Primary ('frailty assessment subgroup pooled estimate') analysis: all data included**<br>Results for all studies (and all frailty tool/outcome dyads) included	Dyads: 16<br>HR: **1.72 (1.46-2.02)**<br>I^2: 46%	Dyads: 21<br>OR: **2.44 (1.55-3.84)**<br>I2: 88%	Dyads: 14<br>OR: **1.79 (1.41-2.27)**<br>I^2: 48%	Dyads: 7<br>OR: **2.02 (1.46-2.79)**<br>I^2: 0%
**Secondary ('overall pooled estimate') meta-analysis:**	Studies: 15<br>HR: **1.68 (1.41-2.00)**	Studies: 17<br>OR: **1.83 (1.24-2.68)**	Studies: 12<br>OR: **1.68 (1.32-2.12)**	Studies: 5<br>OR: **1.94 (1.32-2.83)**

**Multiple reports accounted for***	I^2: 46%	I^2: 76%	I^2: 46%	I^2: 0%
**Sensitivity analysis #1: Studies with low/moderate RoB only***<br>Studies with high oveall RoB on QUIPs removed	Studies: 13<br>HR: **1.65 (1.38-1.97)**<br>I^2: 49%	N/A (nil to remove)	N/A (nil to remove)	N/A (nil to remove)
**Sensitivity analysis #2: Adjusted data only***<br>Studies only reporting univariate analysis (with no adjustment for covariates) removed	Studies: 10<br>HR: **1.95 (1.62-2.35)**<br>I^2: 42%	Studies: 7<br>OR: **1.99 (1.43-2.78)**<br>I^2: 17%	Studies: 5<br>OR: **2.18 (1.76-2.70)**<br>I^2: 0%	N/A (<2 studies)
**Sensitivity analysis #3: GI cancers only**<br>Studies not focussing exclusively on patients with GI cancer removed	Studies: 5<br>HR: **1.50 (1.23-1.83)**<br>I^2: 12%	Studies: 3<br>OR: 1.29 (0.70-2.38)<br>I^2: 0%	N/A (<2 studies)	N/A (<2 studies)
**Sensitivity analysis #4: Advanced/palliative only**<br>Studies that did not predominantly focus on patients with advanced disease and/or undergoing palliative treatment removed	Studies: 12<br>HR: **1.70 (1.33-2.18)**<br>I^2: 56%	Studies: 11<br>OR: **1.60 (1.09-2.35)**<br>I^2: 36%	Studies: 6<br>OR: **2.25 (1.78-2.84)**<br>I^2: 0%	Studies: 3<br>OR: **2.33 (1.45-3.73)**<br>I^2: 0%

Footnotes: The primary analysis includes all data to provide the main result of this meta-analysis in terms of the pooled estimates for each frailty tool/outcome dyad/subgroup. Where a study contributes more than one frailty tool/point estimate to the meta-analysis, the frailty tool with the highest point estimate is removed from the additional analyses (indicated by*). The secondary ('overall pooled estimate') meta-analysis (with multiple reports accounted for) provides the main result of with regards to the pooled estimate for the overall association between frailty and outcome in this study. Statistically significant results in **bold. Acronyms/abbreviations:** CI = confidence interval (95%), G8 = geriatric-8, GI = gastro-intestinal, HR = hazard ratio, I^2 = measure of statistical heterogeneity, N/A = not applicable, no. = number, OR = odds ratio, RoB = Risk of Bias, QUIPs = Quality of Prognosis Studies in Systematic Reviews, VES-13 = vulnerable elders survey-13

Supplementary Figures: Forest plots for primary meta-analysis (all data)

Reported below are forest plots summarising the primary meta-analyses for each outcome **(Supplementary Figures 1-4)**. These analyses include all data from all studies reporting on the association between binary frailty assessment tools and the respective outcome. This analysis provides the main pooled estimate for each frailty tool sub-group. However, studies which report on multiple frailty measures are over-represented in the calculation of overall pooled estimates for the association between frailty and outcome overall. For this reason, overall pooled estimates reported in the main manuscript are taken from the secondary analysis which accounts for multiple reports (**Supplementary Methods 4, Supplementary Table 2, Supplementary Figures 5-8**).

Supplementary Figure 1. Forest plot of the association between binary frailty assessment tools and overall survival (primary analysis, all data, stratified by frailty tool)

Study or Subgroup	log[Hazard Ratio]	SE	Weight	Hazard Ratio IV, Random, 95% CI
**5.1.1 CFS frail (≥4) vs not frail (<4)**				
Moth 2020 (adj)	1.4255	0.2939	5.4%	4.16 [2.34, 7.40]
Shah 2022 (adj)	0.4055	0.1206	12.2%	1.50 [1.18, 1.90]
**Subtotal (95% CI)**			**17.6%**	**2.41 [0.89, 6.54]**
Heterogeneity: Tau² = 0.47; Chi² = 10.31, df = 1 (P = 0.001); I² = 90%				
Test for overall effect: Z = 1.73 (P = 0.08)				
**5.1.2 Fried frail (≥3) vs not frail (<3)**				
Ruiz 2019 (unadj)	0.0296	0.3659	4.0%	1.03 [0.50, 2.11]
**Subtotal (95% CI)**			**4.0%**	**1.03 [0.50, 2.11]**
Heterogeneity: Not applicable				
Test for overall effect: Z = 0.08 (P = 0.94)				
**5.1.3 G8 frail (≤14) vs not frail (>14)**				
Banna 2022 (adj)	1.1314	0.3963	3.5%	3.10 [1.43, 6.74]
Beardo 2019 (adj)	1.141	0.5192	2.2%	3.13 [1.13, 8.66]
Hamacher 2023 (adj)	0.3436	0.2399	7.0%	1.41 [0.88, 2.26]
Jespersen 2021 (adj)	0.4055	0.1974	8.5%	1.50 [1.02, 2.21]
Kobayashi 2022 (unadj)	-0.0726	0.3137	5.0%	0.93 [0.50, 1.72]
Pearce 2022 (adj)	0.6152	0.2078	8.1%	1.85 [1.23, 2.78]
Rier 2022 (adj)	0.5068	0.2899	5.5%	1.66 [0.94, 2.93]
Wildiers 2022 (adj)	0.9676	0.4413	2.9%	2.63 [1.11, 6.25]
**Subtotal (95% CI)**			**42.8%**	**1.69 [1.33, 2.13]**
Heterogeneity: Tau² = 0.03; Chi² = 9.48, df = 7 (P = 0.22); I² = 26%				
Test for overall effect: Z = 4.38 (P < 0.0001)				
**5.1.4 GFI frail (≥4) vs not frail (<4)**				
Aaldriks 2016 (adj)	0.571	0.116	12.4%	1.77 [1.41, 2.22]
Hamaker 2014 (unadj)	0.0583	0.2758	5.9%	1.06 [0.62, 1.82]
**Subtotal (95% CI)**			**18.3%**	**1.46 [0.89, 2.37]**
Heterogeneity: Tau² = 0.09; Chi² = 2.94, df = 1 (P = 0.09); I² = 66%				
Test for overall effect: Z = 1.51 (P = 0.13)				
**5.1.5 VES13 frail (≥3) vs not frail (<3)**				
Feliu 2021 (unadj)	0.4637	0.2315	7.3%	1.59 [1.01, 2.50]
Jespersen 2021 (adj)	0.7419	0.1647	10.0%	2.10 [1.52, 2.90]
Ramsdale 2013 (adj)	2.7479	2.1442	0.2%	15.61 [0.23, 1043.62]
**Subtotal (95% CI)**			**17.4%**	**1.93 [1.48, 2.51]**
Heterogeneity: Tau² = 0.00; Chi² = 1.91, df = 2 (P = 0.38); I² = 0%				
Test for overall effect: Z = 4.90 (P < 0.00001)				
**Total (95% CI)**			**100.0%**	**1.72 [1.46, 2.02]**
Heterogeneity: Tau² = 0.04; Chi² = 27.61, df = 15 (P = 0.02); I² = 46%				
Test for overall effect: Z = 6.45 (P < 0.00001)				
Test for subgroup differences: Chi² = 3.61, df = 4 (P = 0.46), I² = 0%				

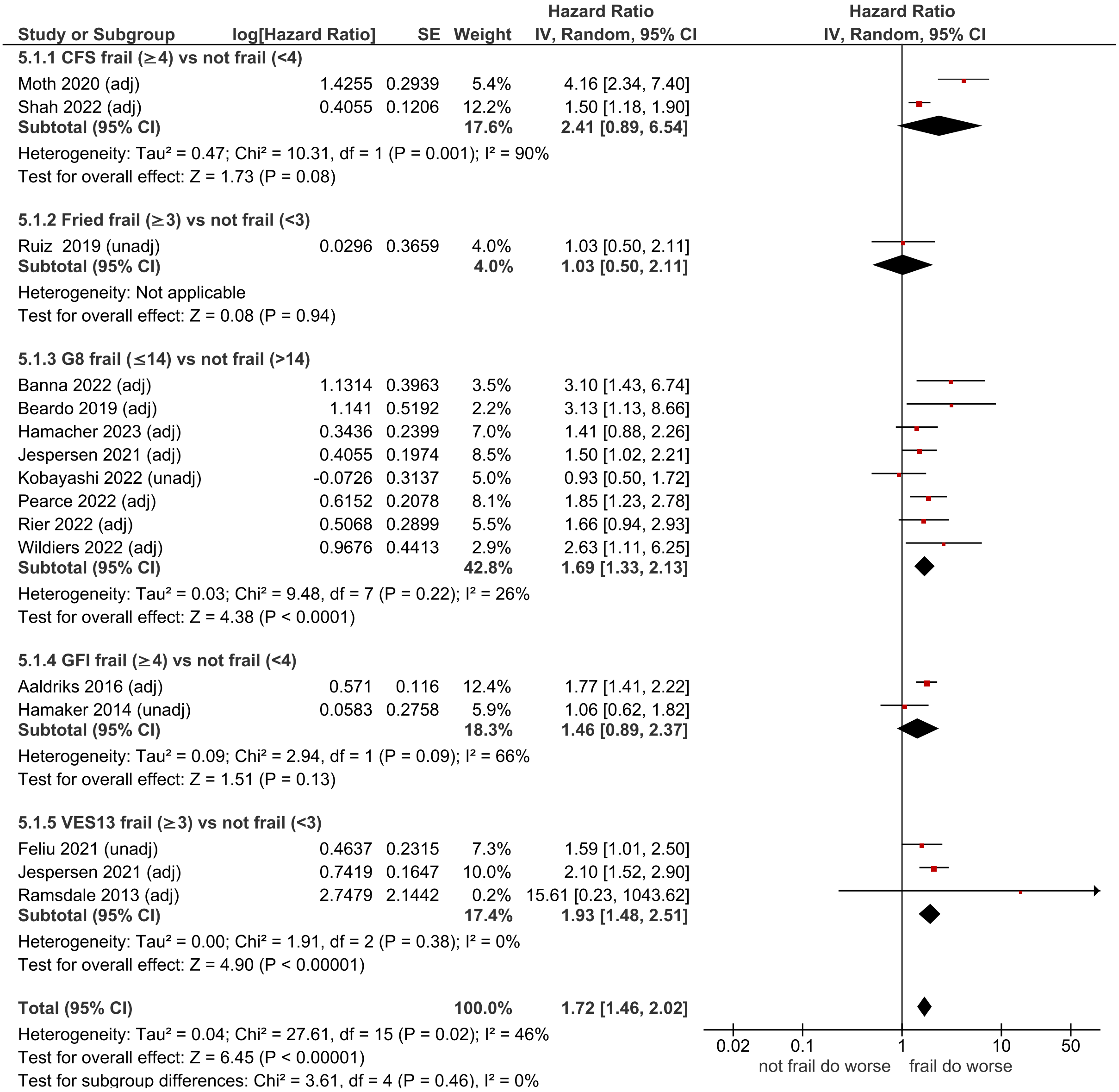

Footnotes:
Hazard ratios relate risk of death in frail patient groups compared with the 'not frail'/'fit' reference category (frailty categories/scores used for each frailty assessment subgroup is provided in the subgroup heading).
For each point estimate, the type of estimate (adjusted vs unadjusted) is specified after the study author/year.
Acronyms/abbreviations: adj = adjusted, CFS = clinical frailty scale, CI = confidence interval, G8 = geriatric-8, GFI = Groningen Frailty Indicator, unadj = unadjusted, VES-13 = vulnerable elders survey-13

Note: Note Ramsdale (2013) is a significant outlier with a large margin of error; it only contributes 0.2% to the pooled estimate for survival and is kept in the forest plot for consistency. Removing the Ramsdale data from this plot does not have a substantial impact on the point estimate/confidence interval (when omitting the Ramsdale data, the pooled estimate from the VES-13 subgroup is 1.91 [1.47-2.49] and the overall pooled estimate is 1.71 [1.45-2.02]).

Supplementary Figure 2. Forest plot of the association between binary frailty assessment tools and grade 3+ toxicity (primary analysis, all data, stratified by frailty tool)

Study or Subgroup	log[Odds Ratio]	SE	Weight	Odds Ratio IV, Random, 95% CI
**5.3.1 Edmonton frail (≥ 7) vs not frail (<7)**				
Akbıyık 2022 (unadj)	-0.9416	0.7073	3.9%	0.39 [0.10, 1.56]
**Subtotal (95% CI)**			**3.9%**	**0.39 [0.10, 1.56]**
Heterogeneity: Not applicable				
Test for overall effect: Z = 1.33 (P = 0.18)				
**5.3.2 Fried frail (≥3) vs not frail (<3)**				
Phaibulvatanapong 2018 (adj)	0.1484	0.4622	4.9%	1.16 [0.47, 2.87]
Ruiz 2019 (adj)	1.9502	0.9421	3.0%	7.03 [1.11, 44.55]
Runzer-Colmenares 2020 (unadj)	2.4168	0.2393	5.6%	11.21 [7.01, 17.92]
**Subtotal (95% CI)**			**13.5%**	**4.45 [0.83, 23.72]**
Heterogeneity: Tau² = 1.85; Chi² = 19.00, df = 2 (P < 0.0001); I² = 89%				
Test for overall effect: Z = 1.75 (P = 0.08)				
**5.3.3 G8 frail (≤14) vs not frail (>14)**				
Bruijnen 2022 (unadj)	0.6109	0.5519	4.5%	1.84 [0.62, 5.43]
Cavdar 2022 (adj)	0.7885	0.3862	5.2%	2.20 [1.03, 4.69]
Chan 2021 (unadj)	-0.367	0.2872	5.5%	0.69 [0.39, 1.22]
Gebbia 2021 (unadj)	0	0.8856	3.2%	1.00 [0.18, 5.67]
Kotzerke 2019 (adj)	1.3737	0.6936	3.9%	3.95 [1.01, 15.38]
Nakazawa 2021 (unadj)	0.7178	0.5793	4.4%	2.05 [0.66, 6.38]
Runzer-Colmenares 2020 (unadj)	2.1021	0.2386	5.7%	8.18 [5.13, 13.06]
Von Minckwitz 2015 (adj)	0.5988	0.4387	4.9%	1.82 [0.77, 4.30]
Winther 2019 (adj)	0.077	0.4095	5.1%	1.08 [0.48, 2.41]
Wu 2023 (unadj)	0.1983	0.4346	5.0%	1.22 [0.52, 2.86]
**Subtotal (95% CI)**			**47.3%**	**1.87 [0.98, 3.56]**
Heterogeneity: Tau² = 0.84; Chi² = 53.17, df = 9 (P < 0.00001); I² = 83%				
Test for overall effect: Z = 1.90 (P = 0.06)				
**5.3.4 GFI frail (≥4) vs not frail (<4)**				
Hamaker 2014 (unadj)	0.6831	0.5766	4.4%	1.98 [0.64, 6.13]
**Subtotal (95% CI)**			**4.4%**	**1.98 [0.64, 6.13]**
Heterogeneity: Not applicable				
Test for overall effect: Z = 1.18 (P = 0.24)				
**5.3.5 VES13 frail (≥3) vs not frail (<3)**				
Alibhai 2021 (unadj)	1.1019	0.6704	4.0%	3.01 [0.81, 11.20]
Cavdar 2022 (adj)	2.3086	0.4215	5.0%	10.06 [4.40, 22.98]
Feliu 2020 (unadj)	0.4318	0.1484	5.9%	1.54 [1.15, 2.06]
Luciani 2015 (adj)	0.8713	0.209	5.7%	2.39 [1.59, 3.60]
Runzer-Colmenares 2020 (unadj)	2.6211	0.2601	5.6%	13.75 [8.26, 22.89]
Winther 2019 (adj)	0.3716	0.4862	4.8%	1.45 [0.56, 3.76]
**Subtotal (95% CI)**			**31.0%**	**3.63 [1.59, 8.32]**
Heterogeneity: Tau² = 0.92; Chi² = 65.22, df = 5 (P < 0.00001); I² = 92%				
Test for overall effect: Z = 3.05 (P = 0.002)				
**Total (95% CI)**			**100.0%**	**2.44 [1.55, 3.84]**
Heterogeneity: Tau² = 0.90; Chi² = 168.34, df = 20 (P < 0.00001); I² = 88%				
Test for overall effect: Z = 3.84 (P = 0.0001)				
Test for subgroup differences: Chi² = 8.24, df = 4 (P = 0.08), I² = 51.5%				

Odds Ratio
IV, Random, 95% CI

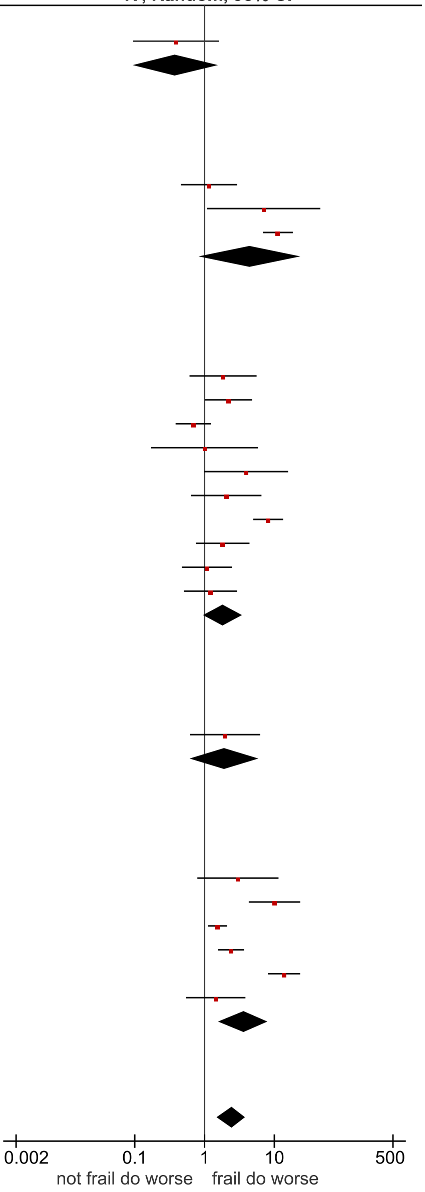

Footnotes:
Odds ratios relate risk of CTCAE grade 3+ toxicity in frail patient groups compared with the 'not frail'/'fit' reference category (frailty categories/scores used for each frailty assessment subgroup is provided in the subgroup heading).
For each point estimate, the type of estimate (adjusted vs unadjusted) is specified after the study author/year.
Acronyms/abbreviations: adj = adjusted, CI = confidence interval, CT CAE = common terminology criteria for adverse events, G8 = geriatric-8, GFI = Groningen Frailty Indicator, unadj = unadjusted, VES-13 = vulnerable elders survey-13

Supplementary Figure 3. Forest plot of the association between binary frailty assessment tools and treatment intolerance (primary analysis, all data, stratified by frailty tool)

Study or Subgroup	log[Odds Ratio]	SE	Weight	Odds Ratio IV, Random, 95% CI
**5.4.1 Fried frail (≥3) vs not frail (<3)**				
Phaibulvatanapong 2018 (unadj)	-0.0943	0.4242	5.5%	0.91 [0.40, 2.09]
**Subtotal (95% CI)**			**5.5%**	**0.91 [0.40, 2.09]**
Heterogeneity: Not applicable				
Test for overall effect: Z = 0.22 (P = 0.82)				
**5.4.2 G8 frail (≤14) vs not frail (>14)**				
Cavdar 2022 (unadj)	1.0367	0.5072	4.3%	2.82 [1.04, 7.62]
Chan 2021 (unadj)	0.5188	0.3686	6.7%	1.68 [0.82, 3.46]
Francolini 2023 (adj)	0.9163	0.1463	14.0%	2.50 [1.88, 3.33]
Hamacher 2023 (adj)	0.8502	0.4804	4.6%	2.34 [0.91, 6.00]
Rier 2022 (adj)	0.8502	0.4187	5.6%	2.34 [1.03, 5.32]
Von Minckwitz 2015 (adj)	0.47	0.3765	6.5%	1.60 [0.76, 3.35]
Winther 2019 (unadj)	1.6014	0.7623	2.2%	4.96 [1.11, 22.10]
Wu 2023 (unadj)	0.0296	0.5011	4.4%	1.03 [0.39, 2.75]
**Subtotal (95% CI)**			**48.3%**	**2.25 [1.81, 2.79]**
Heterogeneity: Tau² = 0.00; Chi² = 5.69, df = 7 (P = 0.58); I² = 0%				
Test for overall effect: Z = 7.28 (P < 0.00001)				
**5.4.3 GFI frail (≥4) vs not frail (<4)**				
Aaldriks 2016 (adj)	0.5188	0.2267	10.9%	1.68 [1.08, 2.62]
**Subtotal (95% CI)**			**10.9%**	**1.68 [1.08, 2.62]**
Heterogeneity: Not applicable				
Test for overall effect: Z = 2.29 (P = 0.02)				
**5.4.4 VES13 frail (≥3) vs not frail (<3)**				
Cavdar 2022 (unadj)	1.1848	0.3773	6.5%	3.27 [1.56, 6.85]
Ferrero 2018 (unadj)	0.5365	0.2098	11.5%	1.71 [1.13, 2.58]
Luciani 2015 (unadj)	0.0198	0.1795	12.7%	1.02 [0.72, 1.45]
Winther 2019 (unadj)	0.6259	0.4863	4.6%	1.87 [0.72, 4.85]
**Subtotal (95% CI)**			**35.3%**	**1.67 [1.03, 2.72]**
Heterogeneity: Tau² = 0.15; Chi² = 9.38, df = 3 (P = 0.02); I² = 68%				
Test for overall effect: Z = 2.08 (P = 0.04)				
**Total (95% CI)**			**100.0%**	**1.79 [1.41, 2.27]**
Heterogeneity: Tau² = 0.08; Chi² = 24.90, df = 13 (P = 0.02); I² = 48%				
Test for overall effect: Z = 4.84 (P < 0.00001)				
Test for subgroup differences: Chi² = 5.64, df = 3 (P = 0.13), I² = 46.8%				

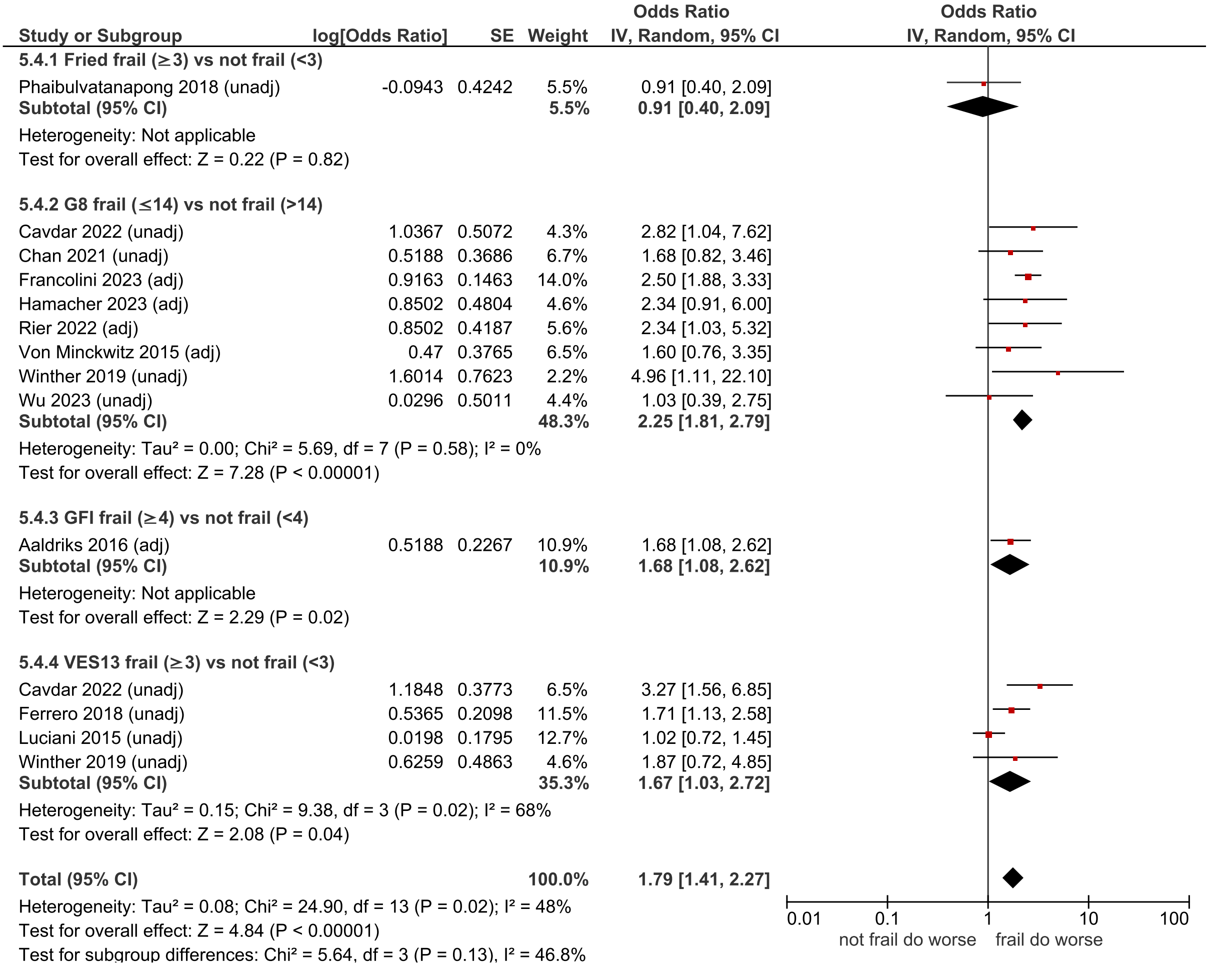

Footnotes:
Odds ratios relate risk of treatment intolerance in frail patient groups compared with the 'not frail'/'fit' reference category (frailty categories/scores used for each frailty assessment subgroup is provided in the subgroup heading).
For each point estimate, the type of estimate (adjusted vs unadjusted) is specified after the study author/year.
Acronyms/abbreviations: adj = adjusted, CI = confidence interval, G8 = geriatric-8, GFI = Groningen Frailty Indicator, unadj = unadjusted, VES-13 = vulnerable elders survey-13

Supplementary Figure 4. Forest plot of the association between binary frailty assessment tools and hospitalisation ((primary analysis, all data, stratified by frailty tool)

Study or Subgroup	log[Odds Ratio]	SE	Weight	Odds Ratio IV, Random, 95% CI
**5.7.1 G8 frail (≤14) vs not frail (>14)**				
Bruijnen 2022 (unadj)	1.0613	0.4776	12.0%	2.89 [1.13, 7.37]
Cavdar 2022 (unadj)	1.0403	0.6353	6.8%	2.83 [0.81, 9.83]
Chan 2021 (unadj)	0.708	0.3104	28.3%	2.03 [1.10, 3.73]
Winther 2019 (adj)	0.3577	0.4126	16.0%	1.43 [0.64, 3.21]
Wu 2023 (unadj)	0.3507	0.4689	12.4%	1.42 [0.57, 3.56]
**Subtotal (95% CI)**			**75.4%**	**1.94 [1.33, 2.81]**
Heterogeneity: Tau² = 0.00; Chi² = 2.06, df = 4 (P = 0.72); I² = 0%				
Test for overall effect: Z = 3.47 (P = 0.0005)				
**5.7.2 VES13 frail (≥3) vs not frail (<3)**				
Cavdar 2022 (unadj)	1.4469	0.489	11.4%	4.25 [1.63, 11.08]
Winther 2019 (adj)	0.2927	0.4552	13.2%	1.34 [0.55, 3.27]
**Subtotal (95% CI)**			**24.6%**	**2.35 [0.76, 7.29]**
Heterogeneity: Tau² = 0.44; Chi² = 2.98, df = 1 (P = 0.08); I² = 66%				
Test for overall effect: Z = 1.48 (P = 0.14)				
**Total (95% CI)**			**100.0%**	**2.02 [1.46, 2.79]**
Heterogeneity: Tau² = 0.00; Chi² = 5.24, df = 6 (P = 0.51); I² = 0%				
Test for overall effect: Z = 4.25 (P < 0.0001)				
Test for subgroup differences: Chi² = 0.10, df = 1 (P = 0.75), I² = 0%				

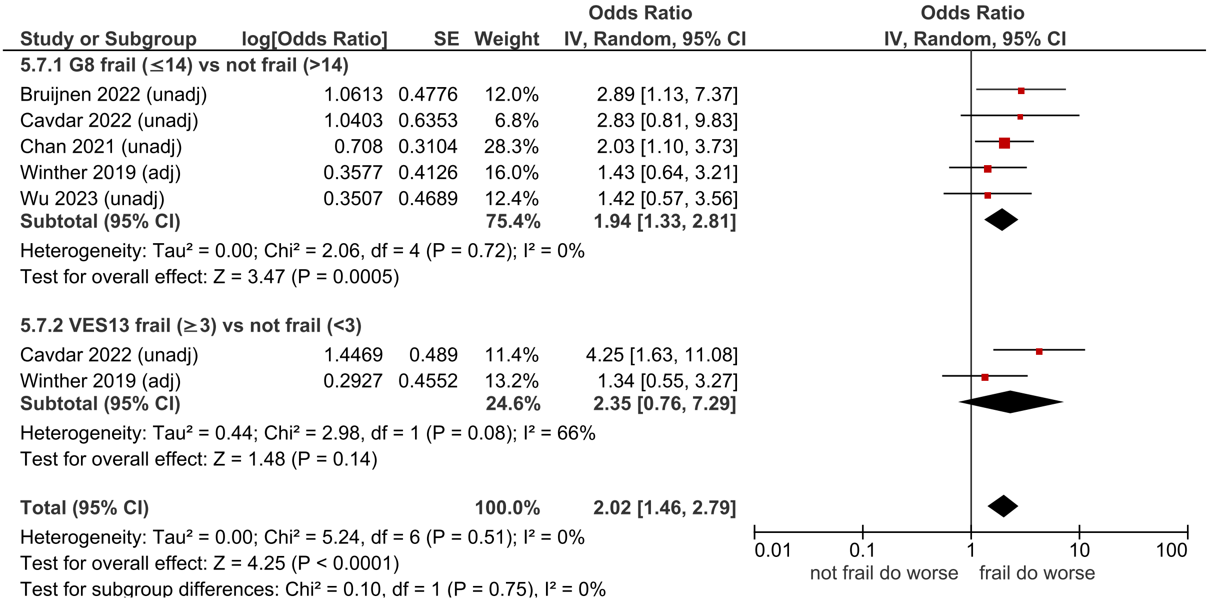

Footnotes:
Odds ratios relate risk of hospitalisation in frail patient groups compared with the 'not frail'/'fit' reference category (frailty categories/scores used for each frailty assessment subgroup is provided in the subgroup heading).
For each point estimate, the type of estimate (adjusted vs unadjusted) is specified after the study author/year.
Acronyms/abbreviations: adj = adjusted, CI = confidence interval, G8 = geriatric-8, unadj = unadjusted, VES-13 = vulnerable elders survey-13

Supplementary Figures: Forest plots for secondary meta-analysis (multiple reports accounted for)

Findings of the secondary meta-analysis which accounts for multiple reports and provides more robust estimates for the overall association between frailty and outcome are provided below.

Supplementary Figure 5. Forest plot of the association between binary frailty assessment tools and overall survival (secondary analysis, multiple reports accounted for)

Study or Subgroup	log[Hazard Ratio]	SE	Weight	Hazard Ratio IV, Random, 95% CI
**5.1.1 CFS frail (≥4) vs not frail (<4)**				
Moth 2020 (adj)	1.4255	0.2939	6.1%	4.16 [2.34, 7.40]
Shah 2022 (adj)	0.4055	0.1206	13.3%	1.50 [1.18, 1.90]
**Subtotal (95% CI)**			**19.3%**	**2.41 [0.89, 6.54]**
Heterogeneity: Tau² = 0.47; Chi² = 10.31, df = 1 (P = 0.001); I² = 90%				
Test for overall effect: Z = 1.73 (P = 0.08)				
**5.1.2 Fried frail (≥3) vs not frail (<3)**				
Ruiz 2019 (unadj)	0.0296	0.3659	4.5%	1.03 [0.50, 2.11]
**Subtotal (95% CI)**			**4.5%**	**1.03 [0.50, 2.11]**
Heterogeneity: Not applicable				
Test for overall effect: Z = 0.08 (P = 0.94)				
**5.1.3 G8 frail (≤14) vs not frail (>14)**				
Banna 2022 (adj)	1.1314	0.3963	4.0%	3.10 [1.43, 6.74]
Beardo 2019 (adj)	1.141	0.5192	2.5%	3.13 [1.13, 8.66]
Hamacher 2023 (adj)	0.3436	0.2399	7.8%	1.41 [0.88, 2.26]
Jespersen 2021 (adj)	0.4055	0.1974	9.5%	1.50 [1.02, 2.21]
Kobayashi 2022 (unadj)	-0.0726	0.3137	5.6%	0.93 [0.50, 1.72]
Pearce 2022 (adj)	0.6152	0.2078	9.0%	1.85 [1.23, 2.78]
Rier 2022 (adj)	0.5068	0.2899	6.2%	1.66 [0.94, 2.93]
Wildiers 2022 (adj)	0.9676	0.4413	3.3%	2.63 [1.11, 6.25]
**Subtotal (95% CI)**			**47.8%**	**1.69 [1.33, 2.13]**
Heterogeneity: Tau² = 0.03; Chi² = 9.48, df = 7 (P = 0.22); I² = 26%				
Test for overall effect: Z = 4.38 (P < 0.0001)				
**5.1.4 GFI frail (≥4) vs not frail (<4)**				
Aaldriks 2016 (adj)	0.571	0.116	13.5%	1.77 [1.41, 2.22]
Hamaker 2014 (unadj)	0.0583	0.2758	6.6%	1.06 [0.62, 1.82]
**Subtotal (95% CI)**			**20.1%**	**1.46 [0.89, 2.37]**
Heterogeneity: Tau² = 0.09; Chi² = 2.94, df = 1 (P = 0.09); I² = 66%				
Test for overall effect: Z = 1.51 (P = 0.13)				
**5.1.5 VES13 frail (≥3) vs not frail (<3)**				
Feliu 2021 (unadj)	0.4637	0.2315	8.1%	1.59 [1.01, 2.50]
Jespersen 2021 (adj)	0.7419	0.1647	0.0%	2.10 [1.52, 2.90]
Ramsdale 2013 (adj)	2.7479	2.1442	0.2%	15.61 [0.23, 1043.62]
**Subtotal (95% CI)**			**8.2%**	**1.84 [0.61, 5.54]**
Heterogeneity: Tau² = 0.28; Chi² = 1.12, df = 1 (P = 0.29); I² = 11%				
Test for overall effect: Z = 1.09 (P = 0.28)				
**Total (95% CI)**			**100.0%**	**1.68 [1.41, 2.00]**
Heterogeneity: Tau² = 0.05; Chi² = 25.72, df = 14 (P = 0.03); I² = 46%				
Test for overall effect: Z = 5.79 (P < 0.00001)				
Test for subgroup differences: Chi² = 2.49, df = 4 (P = 0.65), I² = 0%				

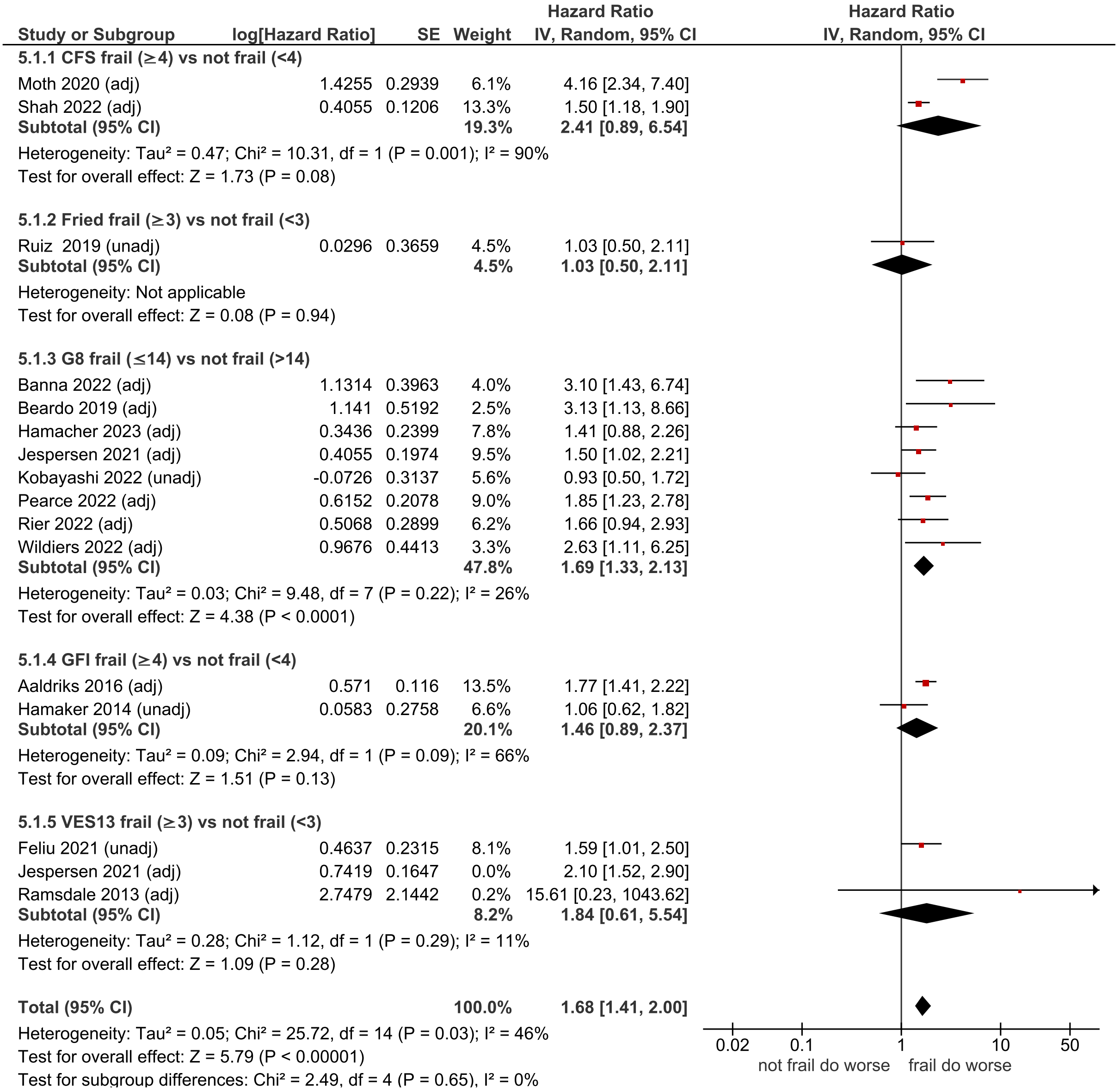

Footnotes:
Hazard ratios relate risk of death in frail patient groups compared with the 'not frail'/'fit' reference category (frailty categories/scores used for each frailty assessment subgroup is provided in the subgroup heading).
For each point estimate, the type of estimate (adjusted vs unadjusted) is specified after the study author/year.
Acronyms/abbreviations: adj = adjusted, CFS = clinical frailty scale, CI = confidence interval, G8 = geriatric-8, GFI = Groningen Frailty Indicator, unadj = unadjusted, VES-13 = vulnerable elders survey-13

Supplementary Figure 6. Forest plot of the association between binary frailty assessment tools and grade 3+ toxicity (secondary analysis, multiple reports accounted for)

Study or Subgroup	log[Odds Ratio]	SE	Weight	Odds Ratio IV, Random, 95% CI
**5.3.1 Edmonton frail (≥ 7) vs not frail (<7)**				
Akbıyık 2022 (unadj)	-0.9416	0.7073	4.2%	0.39 [0.10, 1.56]
**Subtotal (95% CI)**			**4.2%**	**0.39 [0.10, 1.56]**
Heterogeneity: Not applicable				
Test for overall effect: Z = 1.33 (P = 0.18)				
**5.3.2 Fried frail (≥3) vs not frail (<3)**				
Phaibulvatanapong 2018 (adj)	0.1484	0.4622	6.1%	1.16 [0.47, 2.87]
Ruiz 2019 (adj)	1.9502	0.9421	3.0%	7.03 [1.11, 44.55]
Runzer-Colmenares 2020 (unadj)	2.4168	0.2393	0.0%	11.21 [7.01, 17.92]
**Subtotal (95% CI)**			**9.1%**	**2.37 [0.42, 13.32]**
Heterogeneity: Tau² = 1.07; Chi² = 2.95, df = 1 (P = 0.09); I² = 66%				
Test for overall effect: Z = 0.98 (P = 0.33)				
**5.3.3 G8 frail (≤14) vs not frail (>14)**				
Bruijnen 2022 (unadj)	0.6109	0.5519	5.3%	1.84 [0.62, 5.43]
Cavdar 2022 (adj)	0.7885	0.3862	6.8%	2.20 [1.03, 4.69]
Chan 2021 (unadj)	-0.367	0.2872	7.7%	0.69 [0.39, 1.22]
Gebbia 2021 (unadj)	0	0.8856	3.2%	1.00 [0.18, 5.67]
Kotzerke 2019 (adj)	1.3737	0.6936	4.3%	3.95 [1.01, 15.38]
Nakazawa 2021 (unadj)	0.7178	0.5793	5.1%	2.05 [0.66, 6.38]
Runzer-Colmenares 2020 (unadj)	2.1021	0.2386	8.1%	8.18 [5.13, 13.06]
Von Minckwitz 2015 (adj)	0.5988	0.4387	6.3%	1.82 [0.77, 4.30]
Winther 2019 (adj)	0.077	0.4095	6.6%	1.08 [0.48, 2.41]
Wu 2023 (unadj)	0.1983	0.4346	6.4%	1.22 [0.52, 2.86]
**Subtotal (95% CI)**			**60.0%**	**1.87 [0.98, 3.56]**
Heterogeneity: Tau² = 0.84; Chi² = 53.17, df = 9 (P < 0.00001); I² = 83%				
Test for overall effect: Z = 1.90 (P = 0.06)				
**5.3.4 GFI frail (≥4) vs not frail (<4)**				
Hamaker 2014 (unadj)	0.6831	0.5766	5.1%	1.98 [0.64, 6.13]
**Subtotal (95% CI)**			**5.1%**	**1.98 [0.64, 6.13]**
Heterogeneity: Not applicable				
Test for overall effect: Z = 1.18 (P = 0.24)				
**5.3.5 VES13 frail (≥3) vs not frail (<3)**				
Alibhai 2021 (unadj)	1.1019	0.6704	4.4%	3.01 [0.81, 11.20]
Cavdar 2022 (adj)	2.3086	0.4215	0.0%	10.06 [4.40, 22.98]
Feliu 2020 (unadj)	0.4318	0.1484	8.8%	1.54 [1.15, 2.06]
Luciani 2015 (adj)	0.8713	0.209	8.4%	2.39 [1.59, 3.60]
Runzer-Colmenares 2020 (unadj)	2.6211	0.2601	0.0%	13.75 [8.26, 22.89]
Winther 2019 (adj)	0.3716	0.4862	0.0%	1.45 [0.56, 3.76]
**Subtotal (95% CI)**			**21.6%**	**1.93 [1.33, 2.79]**
Heterogeneity: Tau² = 0.05; Chi² = 3.53, df = 2 (P = 0.17); I² = 43%				
Test for overall effect: Z = 3.48 (P = 0.0005)				
**Total (95% CI)**			**100.0%**	**1.83 [1.24, 2.68]**
Heterogeneity: Tau² = 0.41; Chi² = 66.28, df = 16 (P < 0.00001); I² = 76%				
Test for overall effect: Z = 3.08 (P = 0.002)				
Test for subgroup differences: Chi² = 4.94, df = 4 (P = 0.29), I² = 19.1%				

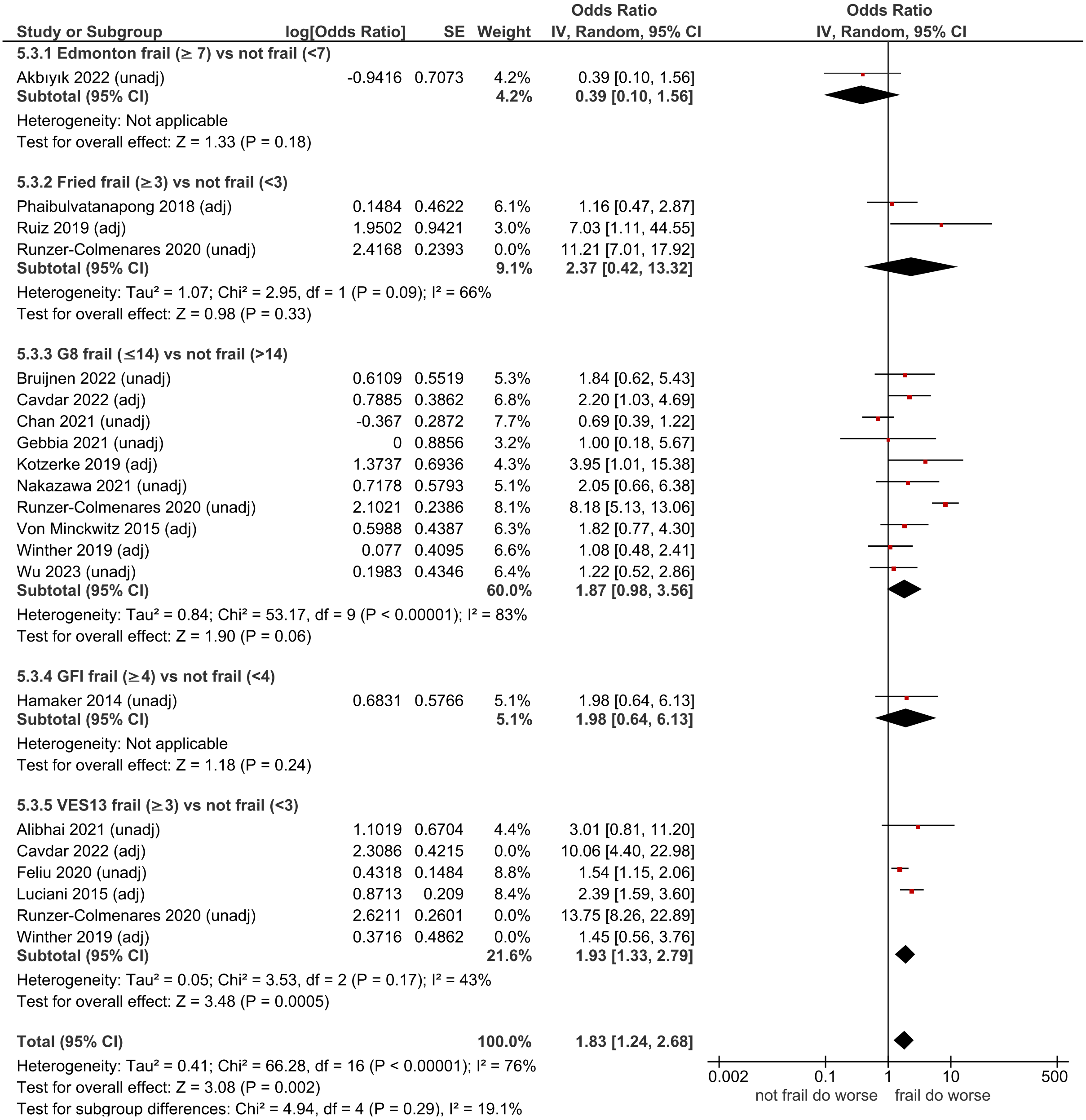

Footnotes:
Odds ratios relate risk of CTCAE grade 3+ toxicity in frail patient groups compared with the 'not frail'/'fit' reference category (frailty categories/scores used for each frailty assessment subgroup is provided in the subgroup heading).
For each point estimate, the type of estimate (adjusted vs unadjusted) is specified after the study author/year.
Acronyms/abbreviations: adj = adjusted, CI = confidence interval, CTCAE = common terminology criteria for adverse events, G8 = geriatric-8, GFI = Groningen Frailty Indicator, unadj = unadjusted, VES-13 = vulnerable elders survey-13

Supplementary Figure 7. Forest plot of the association between binary frailty assessment tools and treatment intolerance (secondary analysis, multiple reports accounted for)

Study or Subgroup	log[Odds Ratio]	SE	Weight	Odds Ratio IV, Random, 95% CI
**5.4.1 Fried frail (≥3) vs not frail (<3)**				
Phaibulvatanapong 2018 (unadj)	-0.0943	0.4242	5.8%	0.91 [0.40, 2.09]
**Subtotal (95% CI)**			**5.8%**	**0.91 [0.40, 2.09]**
Heterogeneity: Not applicable				
Test for overall effect: Z = 0.22 (P = 0.82)				
**5.4.2 G8 frail (≤14) vs not frail (>14)**				
Cavdar 2022 (unadj)	1.0367	0.5072	4.5%	2.82 [1.04, 7.62]
Chan 2021 (unadj)	0.5188	0.3686	7.1%	1.68 [0.82, 3.46]
Francolini 2023 (adj)	0.9163	0.1463	16.2%	2.50 [1.88, 3.33]
Hamacher 2023 (adj)	0.8502	0.4804	4.9%	2.34 [0.91, 6.00]
Rier 2022 (adj)	0.8502	0.4187	6.0%	2.34 [1.03, 5.32]
Von Minckwitz 2015 (adj)	0.47	0.3765	6.9%	1.60 [0.76, 3.35]
Winther 2019 (unadj)	1.6014	0.7623	0.0%	4.96 [1.11, 22.10]
Wu 2023 (unadj)	0.0296	0.5011	4.5%	1.03 [0.39, 2.75]
**Subtotal (95% CI)**			**50.0%**	**2.21 [1.77, 2.75]**
Heterogeneity: Tau² = 0.00; Chi² = 4.58, df = 6 (P = 0.60); I² = 0%				
Test for overall effect: Z = 7.04 (P < 0.00001)				
**5.4.3 GFI frail (≥4) vs not frail (<4)**				
Aaldriks 2016 (adj)	0.5188	0.2267	12.1%	1.68 [1.08, 2.62]
**Subtotal (95% CI)**			**12.1%**	**1.68 [1.08, 2.62]**
Heterogeneity: Not applicable				
Test for overall effect: Z = 2.29 (P = 0.02)				
**5.4.4 VES13 frail (≥3) vs not frail (<3)**				
Cavdar 2022 (unadj)	1.1848	0.3773	0.0%	3.27 [1.56, 6.85]
Ferrero 2018 (unadj)	0.5365	0.2098	12.9%	1.71 [1.13, 2.58]
Luciani 2015 (unadj)	0.0198	0.1795	14.4%	1.02 [0.72, 1.45]
Winther 2019 (unadj)	0.6259	0.4863	4.8%	1.87 [0.72, 4.85]
**Subtotal (95% CI)**			**32.1%**	**1.37 [0.91, 2.07]**
Heterogeneity: Tau² = 0.07; Chi² = 4.09, df = 2 (P = 0.13); I² = 51%				
Test for overall effect: Z = 1.51 (P = 0.13)				
**Total (95% CI)**			**100.0%**	**1.68 [1.32, 2.12]**
Heterogeneity: Tau² = 0.07; Chi² = 20.29, df = 11 (P = 0.04); I² = 46%				
Test for overall effect: Z = 4.28 (P < 0.0001)				
Test for subgroup differences: Chi² = 7.48, df = 3 (P = 0.06), I² = 59.9%				

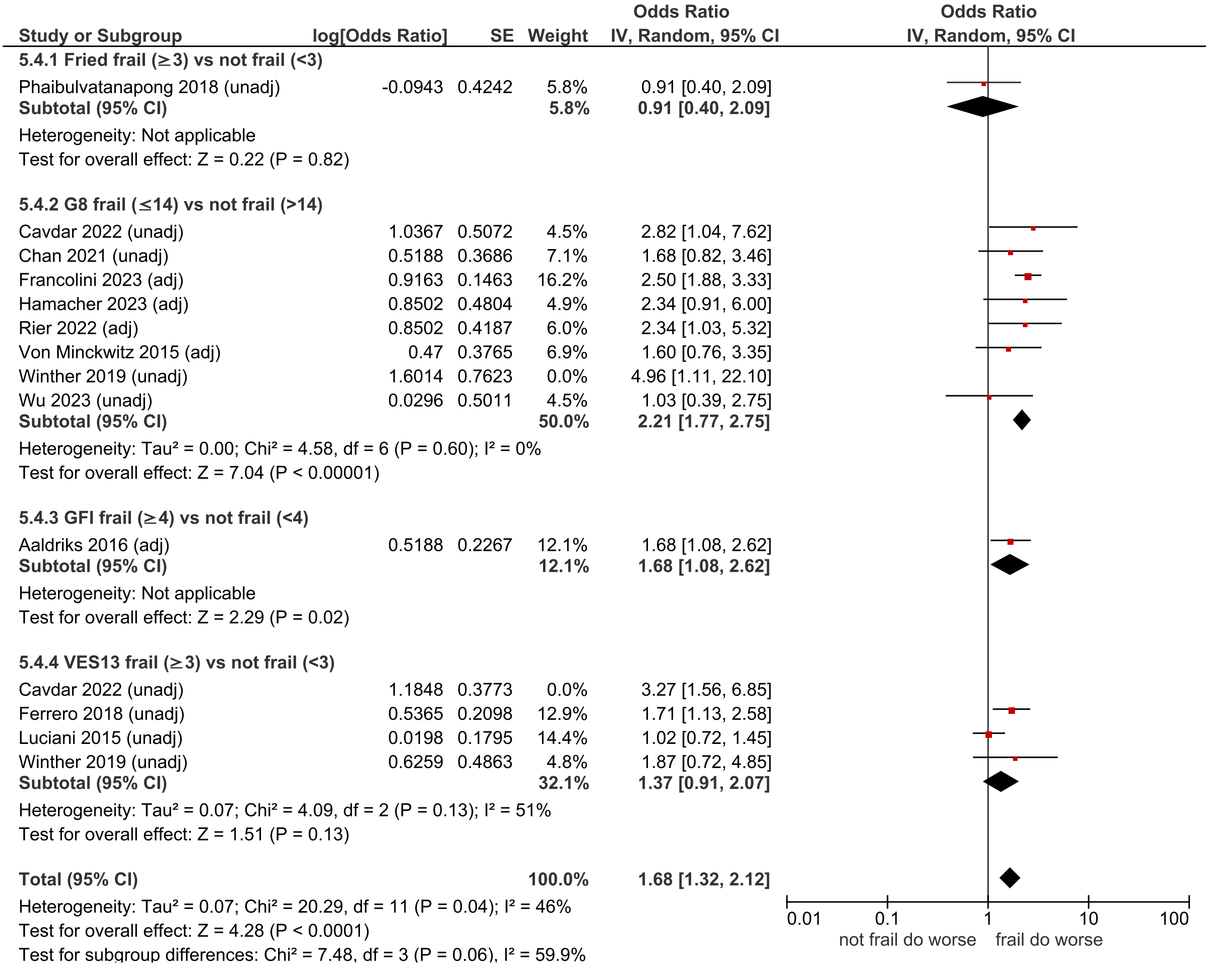

Footnotes:
Odds ratios relate risk of treatment intolerance in frail patient groups compared with the 'not frail'/'fit' reference category (frailty categories/scores used for each frailty assessment subgroup is provided in the subgroup heading).
For each point estimate, the type of estimate (adjusted vs unadjusted) is specified after the study author/year.
Acronyms/abbreviations: adj = adjusted, CI = confidence interval, G8 = geriatric-8, GFI = Groningen Frailty Indicator, unadj = unadjusted, VES-13 = vulnerable elders survey-13

Supplementary Figure 8. Forest plot of the association between binary frailty assessment tools and hospitalisation (secondary analysis, multiple reports accounted for)

Study or Subgroup	log[Odds Ratio]	SE	Weight	Odds Ratio IV, Random, 95% CI
**5.7.1 G8 frail (≤14) vs not frail (>14)**				
Bruijnen 2022 (unadj)	1.0613	0.4776	16.5%	2.89 [1.13, 7.37]
Cavdar 2022 (unadj)	1.0403	0.6353	9.3%	2.83 [0.81, 9.83]
Chan 2021 (unadj)	0.708	0.3104	39.0%	2.03 [1.10, 3.73]
Winther 2019 (adj)	0.3577	0.4126	0.0%	1.43 [0.64, 3.21]
Wu 2023 (unadj)	0.3507	0.4689	17.1%	1.42 [0.57, 3.56]
**Subtotal (95% CI)**			**81.9%**	**2.10 [1.38, 3.20]**
Heterogeneity: Tau² = 0.00; Chi² = 1.38, df = 3 (P = 0.71); I² = 0%				
Test for overall effect: Z = 3.46 (P = 0.0005)				
**5.7.2 VES13 frail (≥3) vs not frail (<3)**				
Cavdar 2022 (unadj)	1.4469	0.489	0.0%	4.25 [1.63, 11.08]
Winther 2019 (adj)	0.2927	0.4552	18.1%	1.34 [0.55, 3.27]
**Subtotal (95% CI)**			**18.1%**	**1.34 [0.55, 3.27]**
Heterogeneity: Not applicable				
Test for overall effect: Z = 0.64 (P = 0.52)				
**Total (95% CI)**			**100.0%**	**1.94 [1.32, 2.83]**
Heterogeneity: Tau² = 0.00; Chi² = 2.17, df = 4 (P = 0.70); I² = 0%				
Test for overall effect: Z = 3.41 (P = 0.0007)				
Test for subgroup differences: Chi² = 0.80, df = 1 (P = 0.37), I² = 0%				

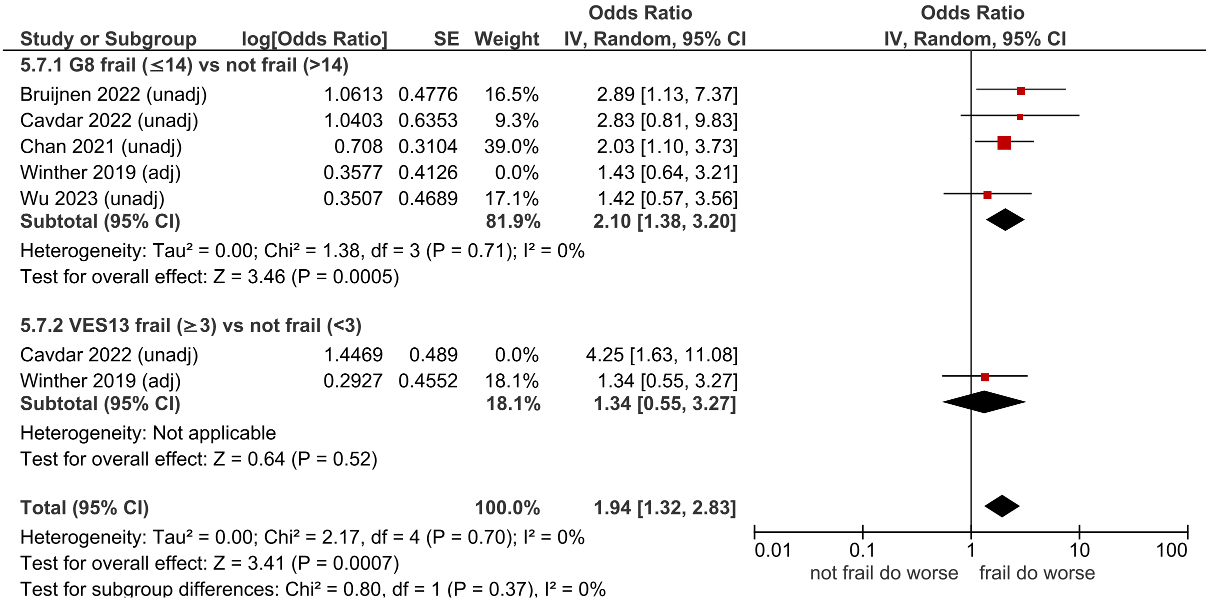

Footnotes:
Odds ratios relate risk of hospitalisation in frail patient groups compared with the 'not frail'/'fit' reference category (frailty categories/scores used for each frailty assessment subgroup is provided in the subgroup heading).
For each point estimate, the type of estimate (adjusted vs unadjusted) is specified after the study author/year.
Acronyms/abbreviations: adj = adjusted, CI = confidence interval, G8 = geriatric-8, unadj = unadjusted, VES-13 = vulnerable elders survey

Supplementary Figures: Funnel plots for primary meta-analysis (all data)

Supplementary Figure 9(a). Funnel plot survival (primary analysis, all data)

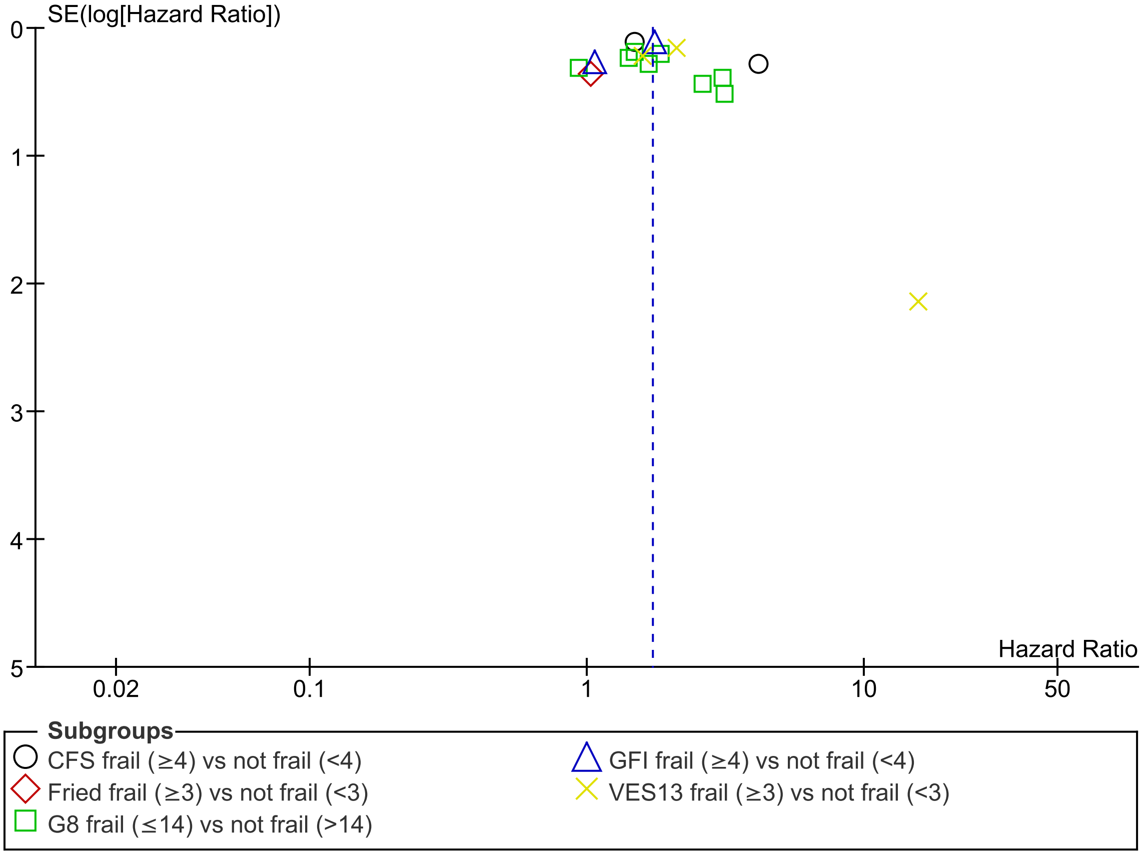

Footnotes:
Acronyms/abbreviations: CFS = clinical frailty scale, G8 = geriatric-8, GFI = Groningen Frailty Indicator, SE = standard error, VES-13 = vulnerable elders survey-13

Note Ramsdale (2013) is a significant outlier with a large margin of error; the additional figure (Supplementary Figure 9(b)) demonstrates the impact of removing this outlier from the funnel plot. It only contributes 0.2% to the pooled estimate for survival and is kept in the forest plot for consistency (removing did not have a substantial impact on the point estimate/confidence interval).

Supplementary Figure 9(b). Funnel plot – survival (primary analysis, Ramsdale 2013 outlier removed)

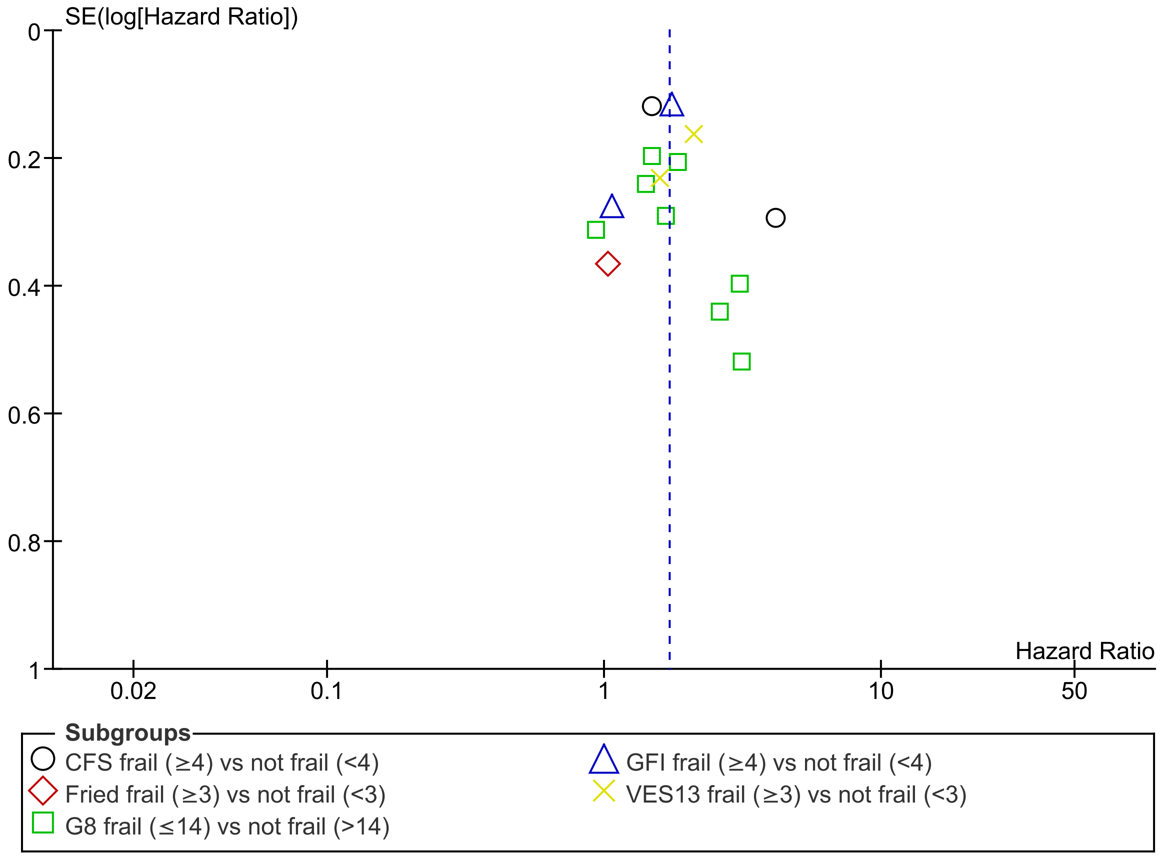

Footnotes:
Acronyms/abbreviations: CFS = clinical frailty scale, G8 = geriatric-8, GFI = Groningen Frailty Indicator, SE = standard error, VES-13 = vulnerable elders survey-13

Supplementary Figure 10. Funnel plot toxicity (primary analysis, all data)

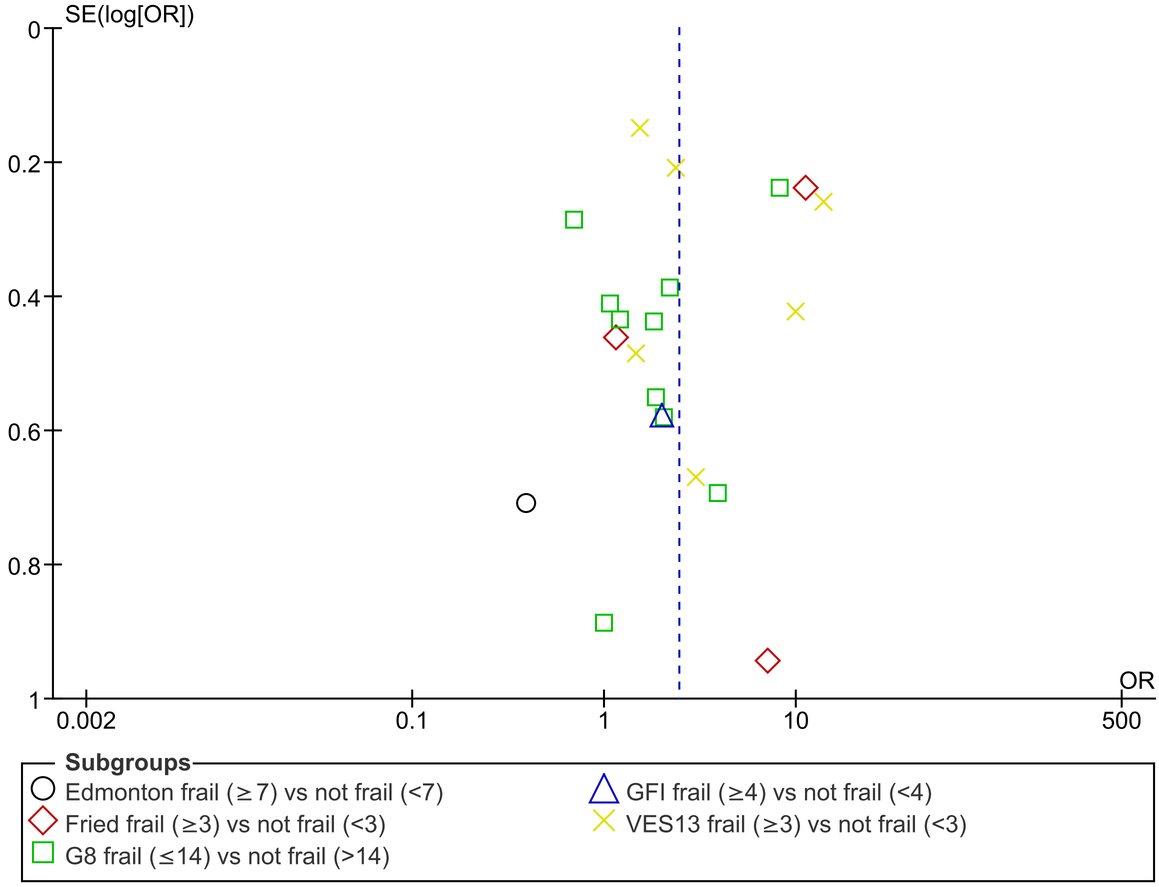

Footnotes:
Acronyms/abbreviations: G8 = geriatric-8, GFI = Groningen Frailty Indicator, OR = odds ratio, SE = standard error, VES-13 = vulnerable elders survey-13

Supplementary Figure 11. Funnel plot treatment intolerance (primary analysis, all data)

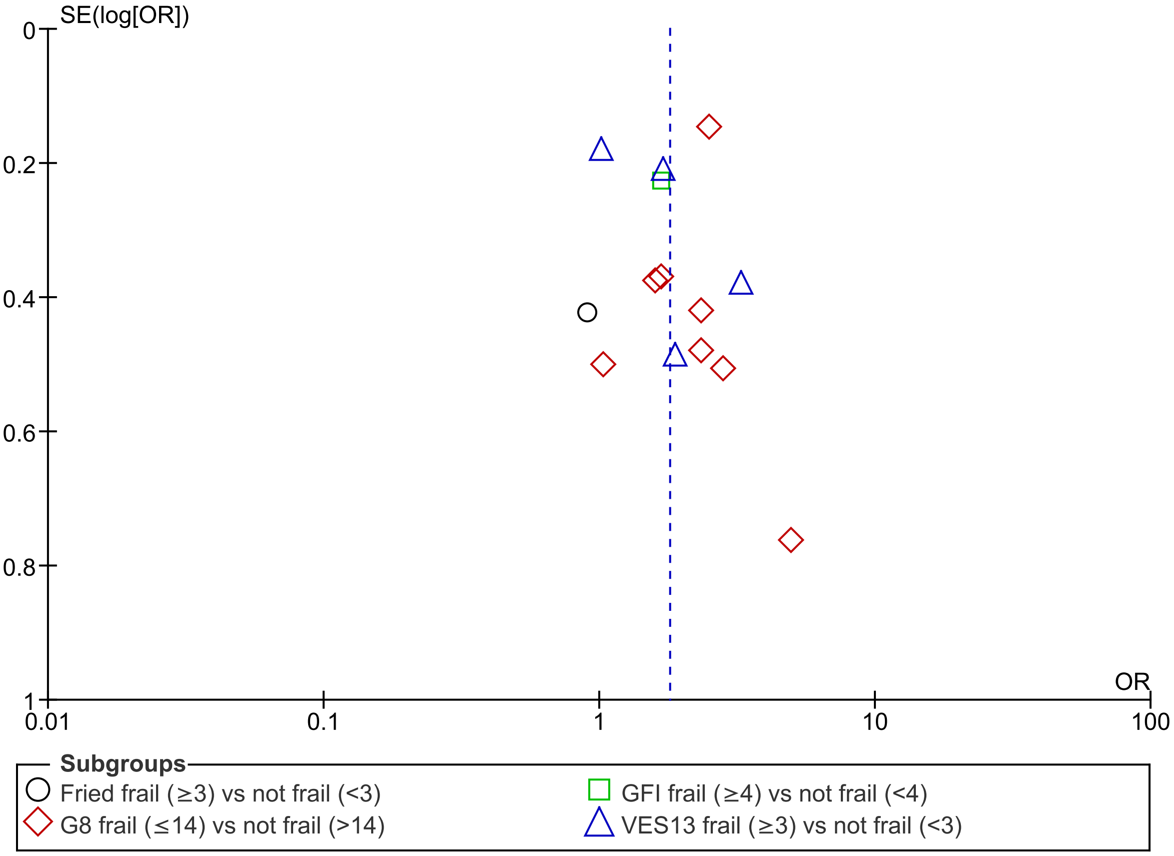

Footnotes:
Acronyms/abbreviations: G8 = geriatric-8, GFI = Groningen Frailty Indicator, OR = odds ratio, SE = standard error, VES-13 = vulnerable elders survey-13

Supplementary Figure 12. Funnel plot hospitalisation (primary analysis, all data)

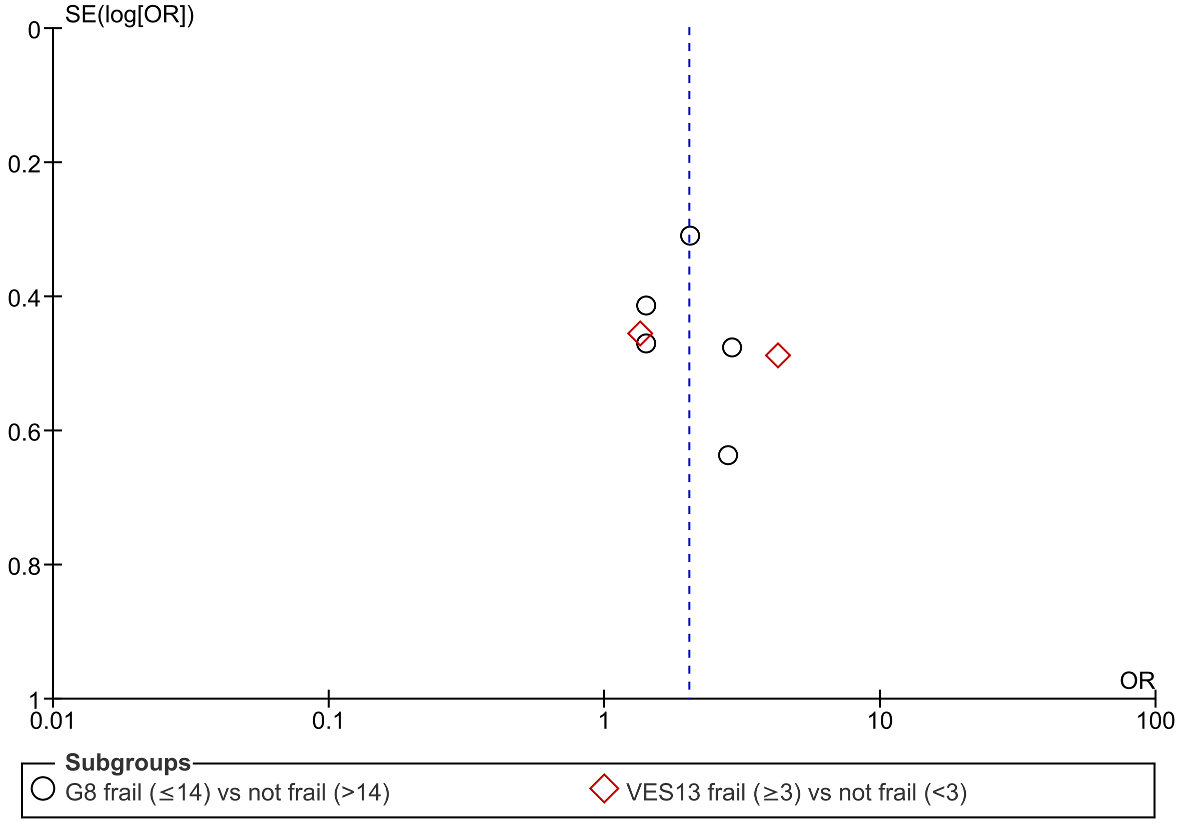

Footnotes:
Acronyms/abbreviations: G8 = geriatric-8, OR = odds ratio, SE = standard error, VES-13 = vulnerable elders survey-13
